# Short-Term Exposure to Ambient Air Pollution and Antimicrobial Use for Acute Respiratory Symptoms

**DOI:** 10.1001/jamanetworkopen.2024.32245

**Published:** 2024-09-06

**Authors:** Gabriela Abelenda-Alonso, Pau Satorra, Marc Marí-Dell’Olmo, Cristian Tebé, Ariadna Padullés, Andrea Vergara, Carlota Gudiol, Miquel Pujol, Jordi Carratalà

**Affiliations:** 1 Department of Infectious Diseases, Bellvitge University Hospital, L’Hospitalet de LLobregat, Barcelona, Catalonia, Spain; 2 Bellvitge Biomedical Research Institute (IDIBELL), L’Hospitalet de Llobregat, Barcelona, Catalonia, Spain; 3 Consortium for Biomedical Research in Infectious Diseases (CIBERINFEC), Instituto de Salud Carlos III, Madrid, Spain; 4 Germans Trias i Pujol Research Institute and Hospital (IGTP), Badalona, Catalonia, Spain; 5 Public Health Agency of Barcelona, Barcelona, Catalonia, Spain; 6 Sant Pau Biomedical Research Institute (IIB Sant Pau), Barcelona, Catalonia, Spain; 7 Consortium for Biomedical Research in the Epidemiology and Public Health Network (CIBERESP), Instituto de Salud Carlos III, Madrid, Spain; 8 Department of Pharmacy, Bellvitge University Hospital, L’Hospitalet de Llobregat, Barcelona, Catalonia, Spain; 9 Department of Microbiology, Hospital Clínic, Barcelona, Catalonia, Spain; 10 Department of Clinical Sciences, Faculty of Medicine and Health Sciences, University of Barcelona, Barcelona, Catalonia, Spain

## Abstract

**Importance:**

Ambient air pollution and antimicrobial resistance pose significant global public health challenges. It is not known whether ambient air pollution is associated with increased consumption of antimicrobials.

**Objective:**

To assess whether a short-term association exists between ambient air pollution levels and antimicrobial consumption among the general population seeking primary care consultations for acute respiratory symptoms.

**Design, Setting, and Participants:**

This 2-stage cross-sectional ecological time series analysis study using data on daily ambient air pollution and antimicrobial consumption was conducted in the 11 largest cities in Catalonia, Spain, from June 23, 2012, to December 31, 2019, among all inhabitants aged 12 years or older. Statistical analysis was performed from November 2022 to December 2023.

**Exposures:**

Daily ambient air pollution (particulate matter of 10 μg/m^3^ [PM_10_], particulate matter of 2.5 μg/m^3^ [PM_2.5_], and nitrogen dioxide [NO_2_]).

**Main Outcomes and Measures:**

The main outcome was antimicrobial consumption associated with primary care consultations for acute respiratory symptoms in the 30 days before and after the dispensing of the antimicrobial. Antimicrobial consumption was measured as defined daily doses (DDDs) per 1000 inhabitants per day.

**Results:**

Among 1 938 333 inhabitants (median age, 48 years [IQR, 34-65 years]; 55% female participants), there were 8 421 404 antimicrobial dispensations, with a median of 12.26 DDDs per 1000 inhabitants per day (IQR, 6.03-15.32 DDDs per 1000 inhabitants per day). The median adjusted morbidity score was 2.0 (IQR, 1.0-5.0). For the 1 924 814 antimicrobial dispensations associated with primary care consultations for acute respiratory symptoms, there was a significant correlation between increases of 10 μg/m^3^ in the concentration of the 3 pollutants studied and heightened antimicrobial consumption at day 0 (PM_10_: relative risk [RR], 1.01 [95% CI, 1.01-1.02]; PM_2.5_: RR, 1.03 [95% CI, 1.01-1.04]; NO_2_: RR, 1.04 [95% CI, 1.03-1.05]). A delayed association emerged between increases in PM_2.5_ concentration and antimicrobial consumption between day 7 (RR, 1.00 [95% CI, 1.00-1.01]) and day 10 (RR, 1.00 [95% CI, 1.00-1.01]) after exposure.

**Conclusions and Relevance:**

In this 2-stage cross-sectional study using ecological time series analysis, short-term exposure to air pollution was associated with increased antimicrobial use associated with primary care consultations for acute respiratory symptoms in the general population. This finding could contribute to informing policy decisions aimed at reducing air pollution and its associated risks, thereby promoting respiratory health and reducing antimicrobial use.

## Key Points

**Question:**

Are short-term exposures to ambient air pollution associated with an increase in antimicrobial use among individuals seeking primary care for acute respiratory symptoms?

**Findings:**

In this 2-stage cross-sectional study using ecological time series analysis, increases in particulate matter of 10 μg/m^3^, particulate matter of 2.5 μg/m^3^ (PM_2.5_), and nitrogen dioxide concentrations were associated with increased antimicrobial consumption on the day of exposure, with a delayed association observed for PM_2.5_ between days 7 and 10 after exposure.

**Meaning:**

These findings suggest that ambient air pollution is associated with increased antimicrobial use for acute respiratory symptoms and underscore the need for more ambitious policies to reduce pollution exposure on a global scale.

## Teaser

This cross-sectional study assesses whether short-term exposures to ambient air pollution are associated with an increase in antimicrobial consumption among the general population seeking primary care consultations for acute respiratory symptoms.

## Introduction

Air pollution and antimicrobial resistance are 2 of the most important public health problems worldwide.^1^ The World Health Organization (WHO) estimates that 95% of the world’s population inhabits places where annual mean air pollution levels surpass the WHO guideline limits and contain high levels of pollutants.^2^ A particular concern is the increase in the global burden of diseases associated with exposure to air pollution, which is now estimated to be responsible for more than 6 million premature deaths every year.^3,4^ Several studies have identified suspended particulate matter of 10 μg/m^3^ (PM_10_) and 2.5 μg/m^3^ (PM_2.5_) as key indicators of air pollution resulting in adverse effects on human health.^5,6,7^ Furthermore, it is widely accepted that long-term exposure to other air pollutants, such as nitrogen dioxide (NO_2_) and tropospheric ozone, even at levels below the current recommended limits, may result in harmful effects on health.^8,9^

Exposure to high levels of air pollution can lead to the development or exacerbation of disorders such as heart disease, stroke, dementia, and lung or breast cancer or chronic and acute respiratory diseases that have been associated with significant increases in primary care consultations.^10,11,12,13^ Another pressing concern is the influence of climate change on the effects of air pollution, which are exacerbated by increasing levels of greenhouse gases. Particularly worrying is the disproportionate effect that climate change has on economically disadvantaged communities.^14^

The WHO has also named antimicrobial resistance as 1 of the 10 greatest global public threats facing humanity. It has been estimated that in 2019, 4.95 million deaths were associated with infections caused by multidrug-resistant bacteria. Moreover, mortality attributable to antimicrobial resistance is expected to reach 10 million deaths per year by 2050.^15^ Crucially, the overuse and misuse of antimicrobial drugs are key drivers of the emergence and dissemination of antimicrobial resistance. In 2018, global antibiotic consumption was estimated at 14.3 defined daily doses (DDDs) per 1000 inhabitants per day, with substantial variations across countries and regions.^16^ In 2021, the mean total antibiotic consumption in Europe was 15.0 DDDs per 1000 inhabitants per day.^17^

Acute respiratory tract infections account for most antimicrobial prescriptions in primary care settings. Although viruses are the leading causal agents of these infections, patients are often inappropriately treated with antibiotics.^18^ Moreover, the onset of acute respiratory symptoms, often triggered by air pollution, can lead to misdiagnosis of respiratory tract infections. Consequently, patients may be prescribed antibiotics unnecessarily, sometimes for extended durations.

Despite the well-established association between air pollution and the occurrence and exacerbation of respiratory diseases, which often leads to increased use of respiratory medications such as bronchodilators, the hypothesis that ambient air pollution may also be associated with heightened antimicrobial use remains unexplored. The present study aimed to investigate whether a short-term correlation exists between ambient air pollution levels and antimicrobial consumption among the general population seeking primary care consultations for acute respiratory symptoms.

## Methods

### Design, Setting, and Population

We performed a 2-stage cross-sectional ecological time series analysis study using data on daily ambient air pollution (PM_10_, PM_2.5_, and NO_2_) and antimicrobial consumption associated with primary care consultations for acute respiratory symptoms in the 30 days before and after the dispensing of the antimicrobial (the ONAIR study). We defined the exposure day (day 0) for ambient air pollutants (PM_10_, PM_2.5_, and NO_2_) as the day when an increase of 10 μg/m^3^ was observed. In the first stage of the analysis, for each city, we examined the association between air pollutants and antimicrobial consumption on the day of exposure to a 10-μg/m^3^ increase in the air pollutant (day 0) and up to 14 days thereafter. In the second stage of the analysis, we pooled these city-specific data using meta-analysis to obtain the aggregated estimates. Antimicrobial consumption was measured as DDDs per 1000 inhabitants per day.^19^ The study was conducted from June 23, 2012, to December 31, 2019, and included inhabitants aged 12 years or older residing in the 11 most populous cities in Catalonia (northeastern Spain), all of which have populations exceeding 100 000 inhabitants (eFigure 1 in Supplement 1). The study was approved by Bellvitge University Hospital Ethics Committee, which waived participant consent because the data were deidentified. The study adhered to the Strengthening the Reporting of Observational Studies in Epidemiology (STROBE) reporting guideline and is registered in the ClinicalTrials.gov database under the identifier NCT04662047.

### Data Collection

We selected participants for the study based on their primary health care service area. Our selection process guaranteed anonymization and included checks to prevent inaccurate area assignment by excluding residents who changed their primary care area during the study period. Health data were sourced from the Public Data Analysis for Health Research and Innovation Program, which provides access to health data generated by the public health system of Catalonia in compliance with current data protection laws. The program is managed by the Catalan Agency for Health Quality and Assessment of the Department of Health of the Catalan government and oversees data extraction.^20^

We identified antimicrobial drugs dispensed through electronic prescriptions from primary care physicians within the public health system, distributed via community pharmacies. For our analysis of the association between ambient air pollution and antimicrobial consumption associated with primary care consultations for acute respiratory symptoms, we specifically focused on antimicrobials usually prescribed for such cases (eTable 1 in Supplement 1). Subsequently, we focused on antimicrobial prescriptions for patients who had primary care consultations for acute respiratory symptoms, identified by *International Statistical Classification of Diseases and Related Health Problems, Tenth Revision* codes ranging from J00 to J99 (excluding J90-J94). We selected a period of 30 days before and after the antimicrobial dispensing to ensure capture of all pertinent prescriptions, including those initiated in emergency departments and that subsequently led to primary care visits.

Daily data on PM_10_, PM_2.5_, and NO_2_ from the 11 cities included in the study were sourced from the Atmospheric Pollution Monitoring and Forecasting Network of the Catalan government.^21^ We used data obtained from urban traffic stations, situated in city centers and directly influenced by vehicle traffic emissions, as well as background stations, located away from emission sources and reflecting the ambient contamination levels of the urban environment. Daily mean values of PM_10_, PM_2.5_, and NO_2_ were computed based on their mean concentrations measured in micrograms per cubic meter across all stations within each city. For PM_2.5_, persistent missing data were encountered in all cities except 2. Consequently, the analysis of PM_2.5_ was conducted only for the cities with data available. Details regarding the distribution and characteristics of ambient air pollution stations in each city can be found in eTable 2 in Supplement 1.

In instances where a variable was missing more than 20% of its data, parametric regression imputation was used. This imputation considered seasonal trends (using Fourier series), the day of the week, holidays, and other pollutants within the same city when available. For variables with less than 20% missing data, imputation was based on measurements from neighboring days within the same week, when available. If a variable was missing more than 50% of its data, the city in question was excluded from the analysis. Information regarding the number and percentage of days with missing data for each pollutant before and after imputation can be found in eTable 3 in Supplement 1.

Meteorologic data such as temperature and relative humidity were recorded daily from the Atmospheric Pollution Monitoring and Forecasting Network of the Catalan government. Our analysis incorporated aggregate and anonymized demographic information, including age, sex, body mass index (calculated as weight in kilograms divided by height in meters squared), adjusted morbidity groups, and social income, using the MEDEA Social Deprivation Index.^22^

### Statistical Analysis

Statistical analysis was performed from November 2022 to December 2023. We used quasi-Poisson generalized linear models with distributed lag nonlinear models to investigate the associations between ambient air pollutants (PM_10_, PM_2.5_, and NO_2_) and antimicrobial consumption, quantified as DDDs per 1000 inhabitants dispensed per day, across each of the 11 cities. The quasi-Poisson generalized linear model incorporated several covariates: day of the year, smoothed using a natural cubic spline with 7 *df* per year, to control the long-term trend and temporal seasonality^23,24^; weekday adjustments to address short-term variations over the week; and temperature and relative humidity adjustments to mitigate potential nonlinear weather-related confounding effects.

We applied distributed lag nonlinear models in the quasi-Poisson generalized linear model to fit the exposure-response association and the lag-response association between each of the daily changing variables and the antimicrobial drugs dispensed.^25^ Specifically, for each ambient air pollutant, we used a linear function to model the exposure-response association and a natural cubic spline with 3 internal knots evenly spaced on the log scale to model the lag-response curve.

We investigated various time lag intervals (in days) ranging from the day of exposure to a 10-μg/m^3^ increase in the air pollutant (day 0) up to 14 days thereafter. Concerning potential confounding factors such as temperature and humidity, the nonlinear exposure response was characterized using a natural cubic spline with 3 internal knots positioned at the 10th, 75th, and 90th percentiles. In addition, the lag-response curve was modeled using a natural cubic spline with 3 equally spaced internal knots on the log scale, with a maximum lag of 21 days. These specifications were chosen based on the model’s performance assessed by the quasi-likelihood version of the Akaike information criterion, as well as insights gleaned from prior studies on the association of air pollution and meteorologic conditions with health outcomes.^26,27^ Subsequently, we used random-effects models to perform a meta-analysis to pool the estimation obtained for each air pollutant and the daily antimicrobial consumption in each city. Aggregate estimates were calculated for each air pollutant, considering an increase of 10 μg/m^3^, across previously defined lags as well as the overall lag period. These estimates were reported as relative risks (RRs) along with their corresponding 95% CIs. To assess the heterogeneity among city estimates, we used the Cochran *Q* test and the Higgins *I*^2^ estimator. In addition, we performed sensitivity analyses by stratifying the data based on sex, age groups (<65 or ≥65 years), and influenza season. Furthermore, we replicated the statistical model to investigate the association between the 3 pollutants studied and the antibiotics dispensed to patients with primary care consultations for acute respiratory symptoms within a 15-day time frame. The model’s assumptions were validated, and 95% CIs for the estimators were calculated wherever feasible. All *P* values were from 2-sided tests and results were deemed statistically significant at *P* < .05. All analyses were conducted using R, version 4.1.0 (2021-05-18) for Windows (R Project for Statistical Computing). Specifically, the primary R packages used in the analysis were DLNM and mvmeta.

## Results

Between June 23, 2012, and December 31, 2019, a total of 8 421 404 antimicrobial dispensations were identified among a population of 1 938 333 inhabitants (median age, 48 years [IQR, 34-65 years]; 55% female participants and 45% male participants) (eTable 4 in Supplement 1). Their median adjusted morbidity score was 2.0 (IQR, 1.0-5.0), and their median body mass index was 26.9 (IQR, 23.5-30.7). Sixty-two percent of individuals had a low to medium social income (<€18 000 per year [less than US $19 537 per year]). Over the 8-year study period, 162 065 participants (8.3%) died. Detailed demographic characteristics of the study population, stratified by each city, are presented in eTable 4 in Supplement 1.

The Table shows the main characteristics of the 11 cities studied, median concentrations of the 3 pollutants under study, and median DDDs per 1000 inhabitants per day. A graphical time series of DDDs per 1000 inhabitants per day is presented in eFigure 2 in Supplement 1. The median global antimicrobial consumption, measured as DDDs per 1000 inhabitants dispensed, was 12.26 (IQR, 6.03-15.32), ranging between 10.24 (IQR, 4.65-12.46) and 14.16 (IQR, 6.89-17.46) across the 11 cities under study (Table).

**Table.** Main Characteristics of the 11 Cities Studied, Median Concentrations of the 3 Pollutants Under Study, and Median DDDs per 1000 Inhabitants per Day

City	Inhabitants, No.	Surface area, km^2^	Air pollution monitoring stations, No.	Median (IQR) concentration, μg/m^3^			DDDs per 1000 inhabitants, median (IQR), d
				PM_10_	PM_2.5_	NO_2_	
Barcelona	1 636 732	101.35	15	24.06 (19.36-29.33)	14.77 (11.95-18.43)	38.23 (29.62-47.82)	10.87 (4.76-12.98)
L’Hospitalet de Llobregat	264 657	12.40	1	22.88 (18.00-28.18)	NA	33.92 (24.48-44.65)	12.41 (5.64-14.94)
Terrassa	223 011	70.16	3	20.59 (16.42-25.53)	NA	41.08 (31.67-51.17)	14.16 (6.89-17.46)
Badalona	223 006	21.18	3	21.38 (17.00-26.17)	NA	36.27 (26.42-47.50)	12.43 (6.37-15.48)
Sabadell	216 204	37.79	2	24.04 (19.31-29.60)	NA	40.25 (30.92-50.17)	13.03 (5.60-15.93)
Lleida	140 080	212.30	2	22.42 (15.79-30.28)	NA	20.40 (14.92-29.04)	12.25 (6.27-15.18)
Tarragona	135 436	57.88	5	18.12 (13.60-23.12)	10.33 (7.00-14.08)	19.22 (14.27-26.43)	12.25 (6.30-14.78)
Mataró	129 120	22.53	2	17.95 (14.27-22.12)	NA	22.42 (16.04-30.96)	12.65 (6.82-15.45)
Santa Coloma de Gramenet	119 289	7.00	2	24.75 (20.42-30.14)	NA	35.08 (26.62-44.31)	13.80 (6.40-17.06)
Reus	106 084	52.82	1	20.79 (15.47-26.52)	NA	16.21 (10.96-24.38)	13.66 (6.47-16.75)
Girona	101 932	39.12	1	20.23 (15.99-25.83)	NA	28.83 (22.59-36.58)	10.24 (4.65-12.46)
Overall	3 295 551	634.53	37	21.46 (16.71-27.17)	12.70 (9.53-16.83)	30.00 (20.25-41.31)	12.26 (6.03-15.32)

Abbreviations: DDD, defined daily dose; NA, not applicable; NO_2_, nitrogen dioxide; PM_10_, particulate matter of 10 μg/m^3^; PM_2.5_, particulate matter of 2.5 μg/m^3^.

Throughout the study, across all 11 cities, the median concentration of PM_10_ was 21.46 μg/m^3^ (IQR, 16.71-27.17 μg/m^3^), and the median concentration of NO_2_ was 30.00 μg/m^3^ (IQR, 20.25-41.31 μg/m^3^). In the 2 cities where daily measurements of PM_2.5_ were available, the median concentration was 12.70 μg/m^3^ (IQR, 9.53-16.83 μg/m^3^). Descriptive statistics detailing potential confounding factors across the 11 cities during the study period can be found in eTable 5 in Supplement 1. In addition, the geographical location of each air pollution station stratified by pollutant can be found in eFigure 3 in Supplement 1.

We identified a total of 1 924 814 antimicrobial dispensations associated with primary care consultations for acute respiratory symptoms. The study flowchart is shown in eFigure 4 in Supplement 1.

**Figure 1.**
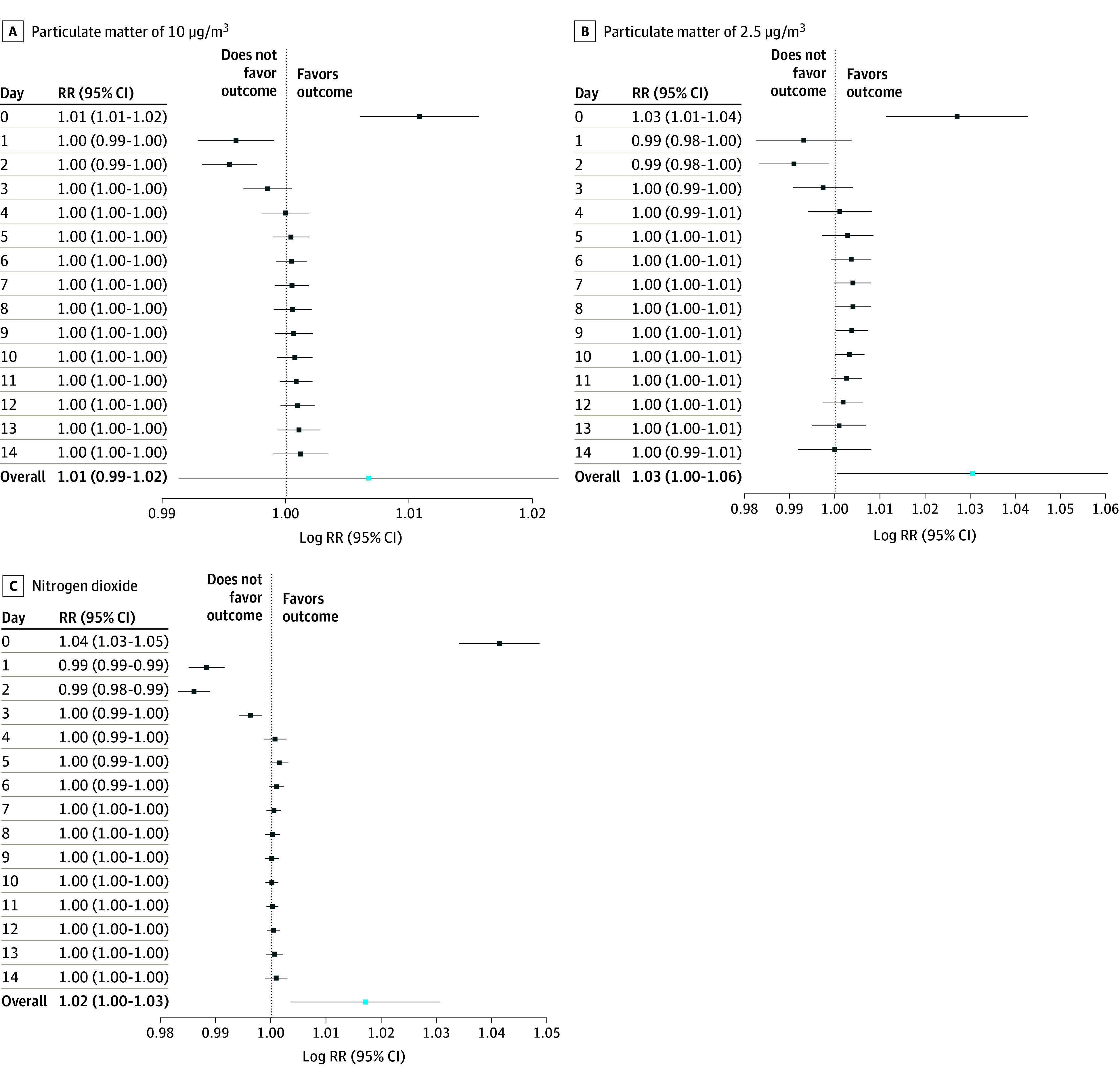
Meta-Analysis of the Overall Estimated Risk of Increased Antimicrobial Consumption With Increases of 10 μg/m^3^ for Particulate Matter of 10 μg/m^3^ (PM_10_), Particulate Matter of 2.5 μg/m^3^ (PM_2.5_)_,_ and Nitrogen Dioxide (NO_2_) Estimated relative risks (RRs) for an exposition to a 10-μg/m^3^ increase in air pollution on the day of exposure (day 0) to each pollutant (PM_10_, PM_2.5_, and NO_2_) and up to 14 days later, with 95% CIs. The overall estimate for the whole lag period is shown. The labels “Does not favor outcome” and “Favors outcome” both refer to antimicrobial use, with the outcome indicating an increase in antimicrobial consumption. The dashed vertical line indicates an RR of 1, corresponding to no association. The x-axis is logarithmically scaled.

**Figure 2.**
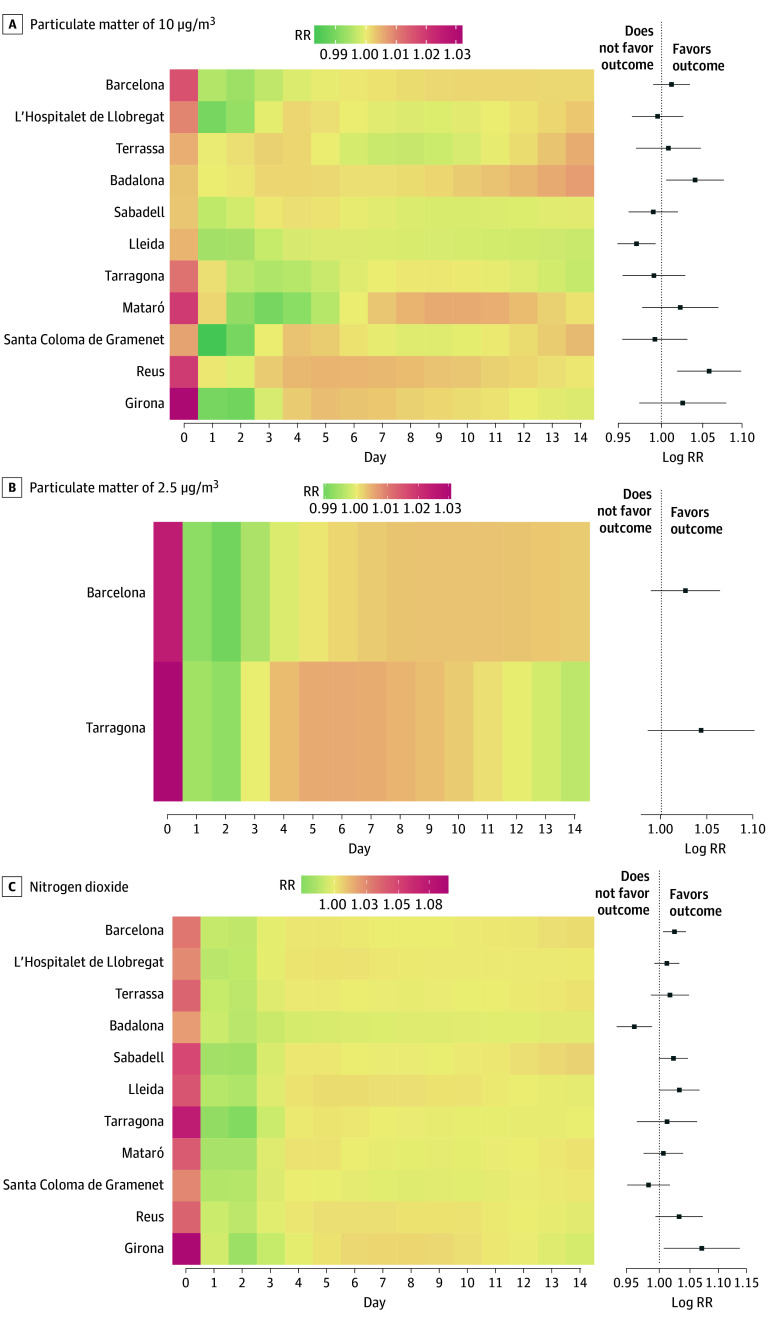
Heatmap for Estimated Risks of Increased Antimicrobial Consumption With an Increase of 10 μg/m^3^ in the Particulate Matter of 10 μg/m^3^ (PM_10_), Particulate Matter of 2.5 μg/m^3^ (PM_2.5_)_,_ and Nitrogen Dioxide (NO_2_) for Each City Heatmaps display the magnitude of the estimated relative risk (RR) for an exposition to a 10-μg/m^3^ increase in air pollution on the day of exposure (day 0) to each pollutant (PM_10_, PM_2.5_, and NO_2_) and up to 14 days later, for each city. Data for PM_2.5_ (panel B) were available for only 2 cities. The overall RR estimate for the whole lag period is shown for each city, with 95% CIs.

As illustrated in Figure 1A, an increase of 10 μg/m^3^ in the concentration of PM_10_ was associated with a significant increase in antimicrobial consumption due to acute respiratory symptoms on day 0 (RR, 1.01 [95% CI, 1.01-1.02]). Furthermore, we observed a protective association between days 1 and 3, as well as a positive but nonsignificant association between day 9 (RR, 1.00 [95% CI, 1.00-1.00]) and day 12 (RR, 1.00 [95% CI, 1.00-1.00]) after exposure. This delayed association varied across the cities studied, as depicted in Figure 2A. Detailed data regarding the meta-analysis of the association of this pollutant for each city can be found in eTable 6 in Supplement 1.

As depicted in Figure 1B, an increase of 10 μg/m^3^ in the concentration of PM_2.5_ was associated with a significant increase in antimicrobial dispensations by day 0 (RR, 1.03 [95% CI, 1.01-1.04]). In addition, we observed a significant protective association by day 2 (RR, 0.99 [95% CI, 0.98-1.00]). We identified a significantly delayed association between day 7 (RR, 1.00 [95% CI, 1.00-1.01]) and day 10 (RR, 1.00 [95% CI, 1.00-1.01]). The association between PM_2.5_ concentrations and antimicrobial consumption, stratified for both cities studied, is illustrated in Figure 2B. Moreover, this delayed association was observed slightly earlier (days 5-6) in the city with the lower median concentration of this pollutant. Detailed data regarding the meta-analysis of the association of this pollutant for each city are presented in eTable 7 in Supplement 1.

As shown in Figure 1C, an increase of 10 μg/m^3^ in the concentration of NO_2_ was associated with a significant increase in antimicrobial consumption due to acute respiratory symptoms on day 0 (RR, 1.04 [95% CI, 1.03-1.05]). As in the case of the association observed with PM_2.5_, the association between NO_2_ and antimicrobial consumption was protective on days 1 and 2 after exposure. However, we did not observe a delayed association from day 5 onward. These findings were consistent across all 11 cities studied, as depicted in Figure 2C. Detailed data regarding the meta-analysis of the association of this pollutant for each city can be found in eTable 8 in Supplement 1.

The sensitivity analysis reaffirmed the findings for all 3 pollutants studied. The associations observed for these pollutants remained consistent even when analyzing antibiotics dispensed to patients within 15 days of primary care consultation (eTable 9 in Supplement 1). Furthermore, factors such as being older than 65 years, male sex, and the influenza season did not substantially alter the associations described.

A meta-analysis of the estimated risk of increased antimicrobial consumption, including the IQR for each pollutant, is presented in eFigure 5 in Supplement 1. In addition, eFigure 6 in Supplement 1 features a heatmap depicting the estimated risk of increased antimicrobial consumption, with the IQR for each pollutant and each city.

## Discussion

This 2-stage cross-sectional ecological time series analysis study using daily ambient air pollution data represents the first investigation, to our knowledge, of the short-term association between air pollution and antimicrobial consumption in the general population. Our main finding was that increases of 10 μg/m^3^ in the concentrations of the 3 pollutants under investigation (PM_10_, PM_2.5_, and NO_2_) were associated with significant increases in antimicrobial consumption associated with primary care consultations for acute respiratory symptoms on the day of exposure (day 0). Our study hypothesizes that air pollution may be associated with increases in antibiotic consumption through 2 primary mechanisms. First, air pollution could induce immediate irritation of the respiratory tract, leading to acute respiratory symptoms that prompt health care–seeking behavior and subsequent antibiotic use. Second, air pollution might trigger innate immune responses in the respiratory system, potentially increasing susceptibility to secondary bacterial infections after viral or pollutant-induced irritation. This secondary infection could result in delayed antibiotic use. In addition, the overlapping symptoms between pollution-induced irritation and acute respiratory tract infections may lead health care professionals to prescribe antibiotics even when the infection is likely viral or pollution induced, both immediately and later.

In the case of PM_10_, our results align with prior research indicating that an elevated PM_10_ concentration in the 3 preceding days was associated with a heightened risk of hospital admission due to acute respiratory exacerbations and a heightened risk of mortality.^5,28,29^ This aligns with our observation of the association on day 0, alongside a significant protective association between days 1 and 3 after exposure, which could be interpreted as a harvest effect, as noted in a previous study.^30^ This phenomenon is commonly observed in time series analysis, describing a temporary increase in the rate of the studied disease after a stressor event (in this case, air pollution). Subsequently, there is a period of lower-than-expected disease rates because the most vulnerable individuals, who required antimicrobial therapy, have already received antibiotic prescriptions. We also observed a nonsignificant association between the concentration of this pollutant and antimicrobial consumption between days 5 and 12. This association exhibited significant heterogeneity across cities, suggesting a need for further investigation to explore the potential delayed association of antimicrobial consumption with increased PM_10_ levels.

In our examination of PM_2.5_, we found a significant association between increased PM_2.5_ concentration and antimicrobial consumption on the day of exposure. This immediate association is consistent with previous research that found a short-term association between increased PM_2.5_ concentrations and emergency department visits or hospitalizations due to culture-negative suspected pneumonia.^31^ In addition, we observed a consistent delayed association occurring between 7 and 10 days after exposure. We hypothesized that this delayed association could be due to the smaller particle size of PM_2.5_, which allows the particles to remain suspended in the air for longer periods and potentially to translocate across the pulmonary epithelium, thus slowing the onset of respiratory symptoms. However, the association calculated for PM_2.5_ presented wide 95% CIs, indicating a higher level of uncertainty regarding this specific association. The absence of a significant association may have been due to the limited number of cities with PM_2.5_ concentrations studied (due to systematic missing data in the rest of the cities).

In our analysis of NO_2_, we found a significant increase in antimicrobial consumption due to acute respiratory symptoms on day 0, followed by a protective association on days 1 and 2. We did not observe a significant delayed association from day 5 onward. Previous studies have consistently shown positive associations between short-term exposure to NO_2_ and respiratory symptom–related emergency department visits, mortality due to pneumonia, and overall mortality.^32,33,34^ In addition, in a recent study involving 147 patients admitted with COVID-19–related pneumonia, it was hypothesized that exposure to NO_2_ in the 2 previous weeks might be an independent risk factor for COVID-19–related pneumonia.^35^

### Limitations

Our study has several limitations that should be acknowledged. First, our case selection was restricted to individuals seeking medical assistance solely within the primary care setting in the public health system. Consequently, the generalizability of our findings may be limited to patients treated in similar primary care settings and may not extend to those receiving treatment in hospitals, other health care facilities, or private health care institutions. Second, our study focused exclusively on antibiotics dispensed via electronic prescriptions made by primary care physicians and distributed through community pharmacies. Therefore, we lack data on private prescriptions or individuals purchasing antibiotics without a prescription on the internet.^36^ Third, measurement of PM_2.5_ was performed daily in only 2 of the 11 cities studied. Fourth, our analysis may be affected by unmeasured and residual confounding factors, including confounding due to individual behaviors that might influence the risk of antimicrobial consumption, as well as potential interactions between PM_2.5_ measurements and NO_2_. In addition, although we conducted a descriptive analysis of the socioeconomic status of the studied population, we were unable to adjust the results for this factor because there is no reliable method to ensure that antimicrobial dispensation at pharmacies corresponds to the same neighborhoods where air pollution was measured or primary care was sought. Fifth, diagnostic or coding errors in health data are inevitable in studies of this kind, and the exact effect of these errors is difficult to assess. However, we attempted to mitigate this drawback by selecting only antibiotics that are commonly used for patients with acute respiratory symptoms.

## Conclusions

In this 2-stage cross-sectional study using ecological time series analysis, we found that short-term exposure to ambient air pollution was associated with increased antimicrobial use for acute respiratory symptoms in the general population. The increase in antimicrobial consumption may be associated with antimicrobial resistance and its potential spread through air pollution.^1^ Our findings underscore the need for more comprehensive and ambitious policies to address ambient air pollution on a global scale. Further investigations conducted across diverse geographical regions are also warranted to confirm and expand on our results, thus fostering a deeper understanding of the intricate association between air pollution and antimicrobial consumption.

## Notes

Supplement 1. **eFigure 1.** The 11 Cities Studied in Catalonia (Northeastern Spain) **eTable 1.** List of Antimicrobials Most Often Prescribed During the Study Period **eTable 2.** Type of Air Pollution Stations in the 11 Cities Studied **eTable 3.** Air Pollutant Median Values, Total Determinations Post-Imputation and Total Determinations Pre-imputation for Each City **eTable 4.** Detailed Demographic Characteristics of the Study Population, Stratified by Each City **eFigure 2.** Temporal Series of DDD per 1,000 Inhabitants-Day for the 11 Cities Studied **eTable 5.** Historical Series of Potential Nonlinear Weather-Related Confounding Factors **eFigure 3.** Map of the Studied Area Showing the Geographical Location of Each Station **eFigure 4.** Study Flowchart **eTable 6.** Meta-analysis of the Estimated Relative Risks (RR [95% CI]) of an Overall Increase in Antimicrobial Consumption With a 10 μg per Cubic Meter Increase in PM_10_ for Each City Studied **eTable 7.** Meta-analysis of the Estimated Relative Risks (RR [95% CI]) of an Overall Increase in Antimicrobial Consumption With a 10 μg per Cubic Meter Increase in PM_2.5_ for Each City Studied **eTable 8.** Meta-analysis of the Estimated Relative Risks (RR [95% CI]) of an Overall Increase in Antimicrobial Consumption With a 10 μg per Cubic Meter Increase in NO_2_ for Each City Studied **eTable 9.** Meta-analysis of the Estimated Relative Risks (RR [95% CI]) of an Increase of a 10-μg per Cubic Meter in Studied Pollutants, in Antimicrobial Consumption for Acute Respiratory Symptoms in the 15 days Preceding and Following the Dispensing of the Antimicrobial **eFigure 5.** Meta-analysis of the Estimated Risk of Increased Antimicrobial Consumption, With Interquartile Range (IQR) for Each Pollutant **eFigure 6.** Heatmap for the Estimated Risk of Increased Antimicrobial Consumption, With Interquartile Range (IQR) for Each Pollutant and Each City

Supplement 2.
Data Sharing Statement
