# Supplementary material for: Short-Term Exposure to Ambient Air Pollution and Antimicrobial Use for Acute Respiratory Symptoms
Source: JAMA Netw Open. 2024 Sep 6;7(9):e2432245. doi: 10.1001/jamanetworkopen.2024.32245 (PMC11380104; doi:10.1001/jamanetworkopen.2024.32245)
Supplement: Supplement 1. — eFigure 1. The 11 Cities Studied in Catalonia (Northeastern Spain) eTable 1. List of Antimicrobials Most Often Prescribed During the Study Period eTable 2. Type of Air Pollution Stations in the 11 Cities Studied eTable 3. Air Pollutant Median Values, Total Determinations Post-Imputation and Total Determinations Pre-imputation for Each City eTable 4. Detailed Demographic Characteristics of the Study Population, Stratified by Each City eFigure 2. Temporal Series of DDD per 1,000 Inhabitants-Day for the 11 Cities Studied eTable 5. Historical Series of Potential Nonlinear Weather-Related Confounding Factors eFigure 3. Map of the Studied Area Showing the Geographical Location of Each Station eFigure 4. Study Flowchart eTable 6. Meta-analysis of the Estimated Relative Risks (RR [95% CI]) of an Overall Increase in Antimicrobial Consumption With a 10 μg per Cubic Meter Increase in PM10 for Each City Studied eTable 7. Meta-analysis of the Estimated Relative Risks (RR [95% CI]) of an Overall Increase in Antimicrobial Consumption With a 10 μg per Cubic Meter Increase in PM2.5 for Each City Studied eTable 8. Meta-analysis of the Estimated Relative Risks (RR [95% CI]) of an Overall Increase in Antimicrobial Consumption With a 10 μg per Cubic Meter Increase in NO2 for Each City Studied eTable 9. Meta-analysis of the Estimated Relative Risks (RR [95% CI]) of an Increase of a 10-μg per Cubic Meter in Studied Pollutants, in Antimicrobial Consumption for Acute Respiratory Symptoms in the 15 days Preceding and Following the Dispensing of the Antimicrobial eFigure 5. Meta-analysis of the Estimated Risk of Increased Antimicrobial Consumption, With Interquartile Range (IQR) for Each Pollutant eFigure 6. Heatmap for the Estimated Risk of Increased Antimicrobial Consumption, With Interquartile Range (IQR) for Each Pollutant and Each City [file jamanetwopen-e2432245-s001.pdf]

Supplementary Online Content

Abelenda-Alonso G, Satorra P, Marí-Dell'Olmo M, et al. Short-term exposure to ambient air pollution and antimicrobial use for acute respiratory symptoms. *JAMA Netw Open.* 2024;7(9):e2432245. doi:10.1001/jamanetworkopen.2024.32245

eFigure 1. The 11 Cities Studied in Catalonia (Northeastern Spain)
eTable 1. List of Antimicrobials Most Often Prescribed During the Study Period
eTable 2. Type of Air Pollution Stations in the 11 Cities Studied
eTable 3. Air Pollutant Median Values, Total Determinations Post-Imputation and Total Determinations Pre-imputation for Each City
eTable 4. Detailed Demographic Characteristics of the Study Population, Stratified by Each City
eFigure 2. Temporal Series of DDD per 1,000 Inhabitants-Day for the 11 Cities Studied
eTable 5. Historical Series of Potential Nonlinear Weather-Related Confounding Factors
eFigure 3. Map of the Studied Area Showing the Geographical Location of Each Station
eFigure 4. Study Flowchart
eTable 6. Meta-analysis of the Estimated Relative Risks (RR [95% CI]) of an Overall Increase in Antimicrobial Consumption With a 10 µg per Cubic Meter Increase in PM_{10} for Each City Studied
eTable 7. Meta-analysis of the Estimated Relative Risks (RR [95% CI]) of an Overall Increase in Antimicrobial Consumption With a 10 µg per Cubic Meter Increase in $PM_{2.5}$ for Each City Studied
eTable 8. Meta-analysis of the Estimated Relative Risks (RR [95% CI]) of an Overall Increase in Antimicrobial Consumption With a 10 µg per Cubic Meter Increase in NO_2 for Each City Studied
eTable 9. Meta-analysis of the Estimated Relative Risks (RR [95% CI]) of an Increase of a 10-µg per Cubic Meter in Studied Pollutants, in Antimicrobial Consumption for Acute Respiratory Symptoms in the 15 days Preceding and Following the Dispensing of the Antimicrobial
eFigure 5. Meta-analysis of the Estimated Risk of Increased Antimicrobial Consumption, With Interquartile Range (IQR) for Each Pollutant
eFigure 6. Heatmap for the Estimated Risk of Increased Antimicrobial Consumption, With Interquartile Range (IQR) for Each Pollutant and Each City

This supplementary material has been provided by the authors to give readers additional information about their work.

© 2024 Abelenda-Alonso G et al. *JAMA Network Open.*

eFigure 1. The 11 Cities Studied in Catalonia (Northeastern Spain)*

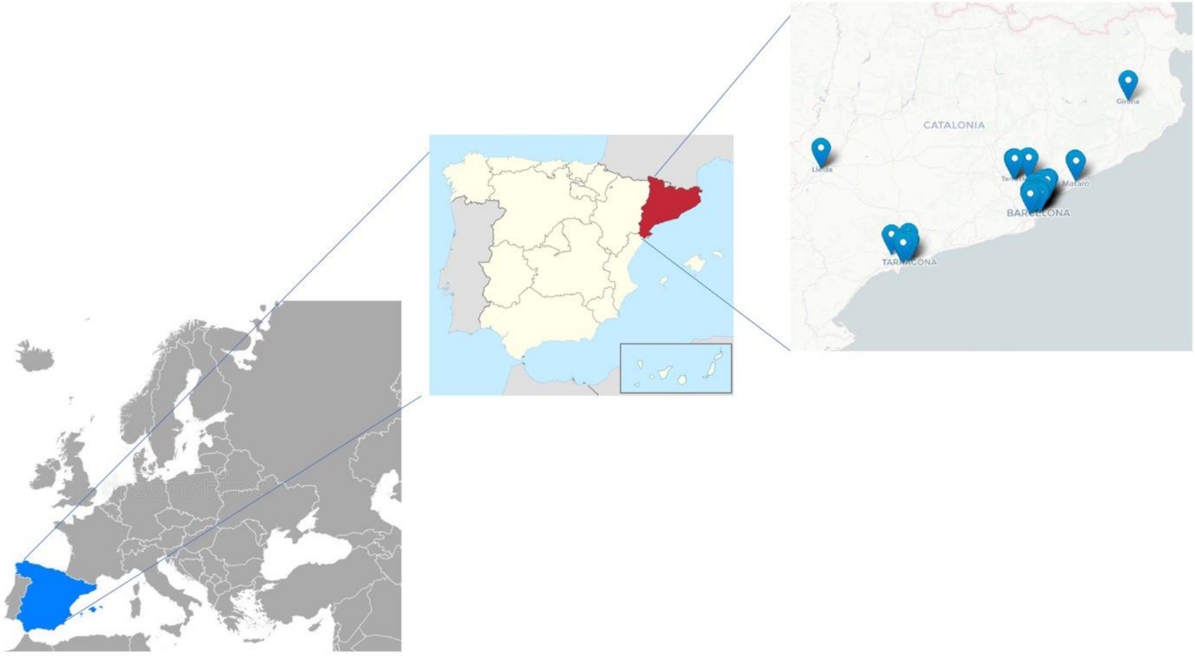

*Catalonia is a populous region sited in the northeast of Spain and is home to 7,522,596 inhabitants. The ONAIR project was carried out from June 23, 2012, to December 31, 2019, and involved inhabitants older than 12 years living in the 11 most populated cities in Catalonia, all with more than 100,000 inhabitants distributed in the region's four provinces. These cities are home to 3,283,501 people.

© 2024 Abelenda-Alonso G et al. *JAMA Network Open.*

eTable 1. List of Antimicrobials Most Often Prescribed During the Study Period

**Antimicrobial**	**Number of dispensations***
Amoxicillin	943,561
Amoxicillin/clavulanic	1,337,507
Azithromycin	673,586
Cefixime	77,673
Cefuroxime	175,768
Ciprofloxacin	468,624
Clarithromycin	120,115
Clindamycin	99,138
Doxycycline	68,809
Levofloxacin	36,1343
Metronidazole	80,783
Trimethoprim-Sulfamethoxazole	86,128

*Total number of dispensed antimicrobials identified by drug regardless of dosage or posology.

© 2024 Abelenda-Alonso G et al. *JAMA Network Open.*

eTable 2. Type of Air Pollution Stations in the 11 Cities Studied

	PM_{10}			$PM_{2.5}$			$N0_2$		
**City**	**Background**	**Traffic station**	**Industrial station**	**Background**	**Traffic station**	**Industrial station**	**Background**	**Traffic station**	**Industrial station**
BARCELONA	5	2	0	-	-	-	5	2	0
L'HOSPITALET DE LLOBREGAT	1	0	0	1	0	0	1	0	0
TERRASA	0	1	0	-	-	-	0	1	0
BADALONA	1	0	0	-	-	-	1	0	0
SABADELL	5	2	0	0	1	0	0	1	0
LLEIDA	0	1	0	0	1	0	0	1	0
TARRAGONA	1	0	0	0	0	2	1	1	2
MATARÓ	1	0	0	1	0	0	1	0	0
SANTA COLOMA DE GRAMANET	0	1	0	1	0	0	1	0	0
REUS	1	0	0	-	-	-	0	1	0
GIRONA	0	1	0	-	-	-	0	1	0

*Background air pollution is defined as the ambient level of pollution that is not affected by local sources of pollution. Traffic air pollution is referred to the mixture of vehicule exhausts, secondary pollutants formed in the atmospherere, evaporative emissions from vehicles and non-combustion emissions. Industrial air pollution is defined as that emitted by industrial facilities.

© 2024 Abelenda-Alonso G et al. *JAMA Network Open.*

eTable 3. Air Pollutant Median Values, Total Determinations Post-Imputation and Total Determinations Pre-imputation for Each City*. The Study Period Includes 2,748 Days

	PM_{10}			$PM_{2.5}$			$N0_2$		
	**Median µg per cubic meter**	**Post-imputation n (%)**	**Pre-imputation n (%)**	**Median µg per cubic-meter**	**Post-imputation n (%)**	**Pre-imputation n (%)**	**Median µg per cubic meter**	**Post-imputation n (%)**	**Pre-imputation n (%)**
BARCELONA	24.06 [19.36; 29.33]	2,748 (100%)	2,748 (100%)	14.77 [11.95; 18.43]	2,748 (100%)	2,748 (100%)	38.23 [29.62; 47.82]	2,748 (100%)	2,748 (100%)
L'HOSPITALET DE LLOBREGAT	23.01 [18; 28.12]	2,704 (98.4%)	2,532 (92.14%)	-	-	1,356 (49.34%)	33.92 [24.48; 44.65]	2,723 (99.09%)	2,698 (98.18%)
TERRASA	21.72 [17.08; 26.23]	2,720 (98.98%)	2,126 (77.37%)	-	-	0 (0%)	41.08 [31.67; 51.17]	2,717 (98.87%)	2,704 (98.4%)
BADALONA	21.47 [17.11; 26.06]	2,734 (99.49%)	2,441 (88.83%)	-	-	0 (0%)	36.27 [26.42; 47.5]	2,690 (97.89%)	2,675 (97.34%)
SABADELL	25 [20.28; 30.29]	2,723 (99.09%)	2,074 (75.47%)	-	-	1,323 (48.14%)	40.25 [30.92; 50.17]	2,715 (98.8%)	2,692 (97.96%)
LLEIDA	22.5 [15.81; 30.28]	2,703 (98.36%)	2,682 (97.6%)	-	-	1,256 (45.71%)	20.4 [14.92; 29.04]	2,693 (98%)	2,626 (95.56%)
TARRAGONA	18.21 [13.6; 23.12]	2,728 (99.27%)	2,667 (97.05%)	10.33 [7; 14.08]	2,742 (99.78%)	2,610 (94.98%)	19.22 [14.27; 26.43]	2,748 (100%)	2,748 (100%)
MATARÓ	17.98 [14.29; 22.12]	2,716 (98.84%)	2,696 (98.11%)	-	-	1,310 (47.67%)	22.42 [16.04; 30.96]	2,702 (98.33%)	2,629 (95.67%)
SANTA COLOMA DE GRAMANET	25.26 [20.72; 30.23]	2,736 (99.56%)	1,314 (47.82%)	-	-	1,286 (46.8%)	35.08 [26.62; 44.31]	2,743 (99.82%)	2,729 (99.31%)
REUS	20.82	2,678	2,592	-	-	0	16.21	2,577	2,516

© 2024 Abelenda-Alonso G et al. *JAMA Network Open.*

	[15.54; 26.47]	(97.45%)	(94.32%)			(0%)	[10.96; 24.38]	(93.78%)	(91.56%)
GIRONA	21.73	2,709	1,768	-	-	0	28.83	2,726	2,657
	[18.17; 26.58]	(98.58%)	(64.34%)			(0%)	[22.59; 36.58]	(99.2%)	(96.69%)

*In instances where a variable was missing more than 20% of its data, parametric regression imputation was employed. This imputation considered seasonal trends (utilizing Fourier series), the day of the week, holidays, and other pollutants within the same city when available. For variables with less than 20% missing data, imputation was based on measurements from neighboring days within the same week, when available. If a variable was missing over 50% of its data, the respective city was excluded from the analysis.

© 2024 Abelenda-Alonso G et al. *JAMA Network Open.*

eTable 4. Detailed Demographic Characteristics of the Study Population, Stratified by Each City

	Overall n=1,938,333	BADALONA n=133,379	BARCELONA n=890,905	GIRONA n=55,265	HOSPITALET DE LLOBREGAT n=162,154	LLEIDA n=100,566	MATARÓ n=77,151	REUS n=68,705	SABADELL n=129,742	SANTA COLOMA DE GRAMENET n=77,843	TARRAGONA n=90,578	TERRASSA n=15,2045
**Sex**												
Men	871,386 (44.96%)	61,465 (46.08%)	39,0341 (43.81%)	24,581 (44.48%)	73,478 (45.31%)	47,402 (47.14%)	35,753 (46.34%)	31,655 (46.07%)	59,185 (45.62%)	36,425 (46.79%)	41,572 (45.90%)	69,529 (45.73%)
Women	1,066,947 (55.04%)	71,914 (53.92%)	50,0564 (56.19%)	30,684 (55.52%)	88,676 (54.69%)	53,164 (52.86%)	41,398 (53.66%)	37,050 (53.93%)	70,557 (54.38%)	41,418 (53.21%)	49,006 (54.10%)	82,516 (54.27%)
**Age**												
Mean (SD)	49.71 (19.49)	47.43 (17.71)	50.81 (19.63)	47.76 (19.80)	49.94 (19.65)	48.82 (19.57)	49.04 (19.43)	48.80 (19.52)	49.70 (19.84)	48.99 (19.21)	48.38 (19.24)	48.23 (19.39)
Median (IQR)	48.00 (34.00, 65.00)	46.00 (33.00, 62.00)	49.00 (35.00, 67.00)	45.00 (32.00, 62.00)	48.00 (34.00, 66.00)	46.00 (34.00, 63.00)	47.00 (34.00, 64.00)	47.00 (33.00, 63.00)	48.00 (34.00, 65.00)	47.00 (34.00, 65.00)	46.00 (33.00, 63.00)	46.00 (33.00, 63.00)
**Income (per year)**												
Poverty threshold	88,859 (4.6%)	7,313 (5.5%)	35,724 (4.0%)	2,887 (5.2%)	6,905 (4.3%)	5,664 (5.6%)	4,201 (5.4%)	4,460 (6.5%)	5,980 (4.6%)	3,807 (4.9%)	4,675 (5.2%)	7,243 (4.8%)

© 2024 Abelenda-Alonso G et al. *JAMA Network Open.*

<18,000€	1,209,206 (62%)	86,016 (64%)	523,302 (59%)	33,538 (61%)	111,570 (69%)	67,359 (67%)	52,912 (69%)	45,091 (66%)	84,924 (65%)	54,790 (70%)	53,893 (59%)	95,811 (63%)
18,001 – 100,000€	624,382 (32%)	39,578 (30%)	320,553 (36%)	18,365 (33%)	43,377 (27%)	27,074 (27%)	19,761 (26%)	18,831 (27%)	38,245 (29%)	19,167 (25%)	31,405 (35%)	48,026 (32%)
>100,000€	15,886; (0.8%)	472 (0.4%)	11,326 (1.3%)	475 (0.9%)	302 (0.2%)	469 (0.5%)	277 (0.4%)	323 (0.5%)	593 (0.5%)	79 (0.1%)	605 (0.7%)	965 (0.6%)
**MEDEA Social Deprivation Index**$^{\Psi}$												
Mean (SD)	40.69 (18.66)	55.55 (20.48)	31.43 (17.22)	34.13 (10.73)	53.79 (8.81)	43.67 (9.53)	52.25 (18.01)	48.17 (11.20)	48.20 (17.96)	61.24 (7.49)	42.56 (18.74)	41.05 (12.31)
Median (IQR)	39.30 (27.34, 54.55)	55.47 (41.05, 72.28)	32.20 (18.40, 40.09)	26.25 (26.25, 47.30)	57.04 (47.51, 60.81)	47.15 (37.10, 51.26)	54.55 (34.06, 68.07)	53.11 (42.34, 54.87)	42.32 (39.77, 62.95)	60.08 (53.98, 68.48)	33.80 (29.02, 64.49)	36.22 (35.36, 49.28)
**Body mass index**												
Mean (SD)	27.40 (5.63)	27.45 (5.86)	27.36 (5.48)	26.92 (5.80)	27.87 (5.52)	26.72 (5.53)	27.08 (5.67)	26.68 (6.20)	27.52 (5.64)	27.97 (5.85)	26.87 (5.93)	27.79 (6.15)

© 2024 Abelenda-Alonso G et al. *JAMA Network Open.*

Median (IQR)	26.90 (23.49, 30.68)	27.00 (23.44, 30.78)	26.84 (23.52, 30.54)	26.00 (22.92, 30.00)	27.34 (23.99, 31.11)	26.15 (22.76, 29.96)	26.64 (23.14, 30.47)	26.08 (22.21, 30.86)	27.13 (23.60, 30.82)	27.61 (24.06, 31.27)	26.32 (22.78, 29.98)	27.03 (23.72, 31.25)
**Adjusted morbidity groups***												
Mean (SD)	3.46 (3.95)	3.65 (3.98)	3.42 (3.94)	3.19 (3.69)	3.65 (4.09)	3.00 (3.66)	3.84 (4.9)	3.36 (3.63)	3.45 (3.88)	3.69 (4.18)	3.12 (3.71)	3.73 (4.07)
Median (IQR)	2.00 (1.00, 5.00)	2.00 (1.00, 5.00)	2.00 (1.00, 5.00)	2.00 (1.00, 5.00)	2.00 (1.00, 5.00)	2.00 (0.00, 4.00)	2.00 (1.00, 5.00)	2.00 (1.00, 5.00)	2.00 (1.00, 5.00)	2.00 (1.00, 5.00)	2.00 (1.00, 4.00)	2.00 (1.00, 5.00)

$^{\Psi}$MEDEA social deprivation index was calculated for using information related to five social deprivation indicators related to word and education from the 2001 national census in Spain. *The Adjusted Morbidity Groups is a morbidity measurement developed and adapted to the Spanish healthcare system. It classifies the population into six morbidity groups: one healthy, and five with different levels of complexity.

© 2024 Abelenda-Alonso G et al. *JAMA Network Open.*

eFigure 2. Temporal Series of DDD per 1,000 Inhabitants-Day for the 11 Cities Studied

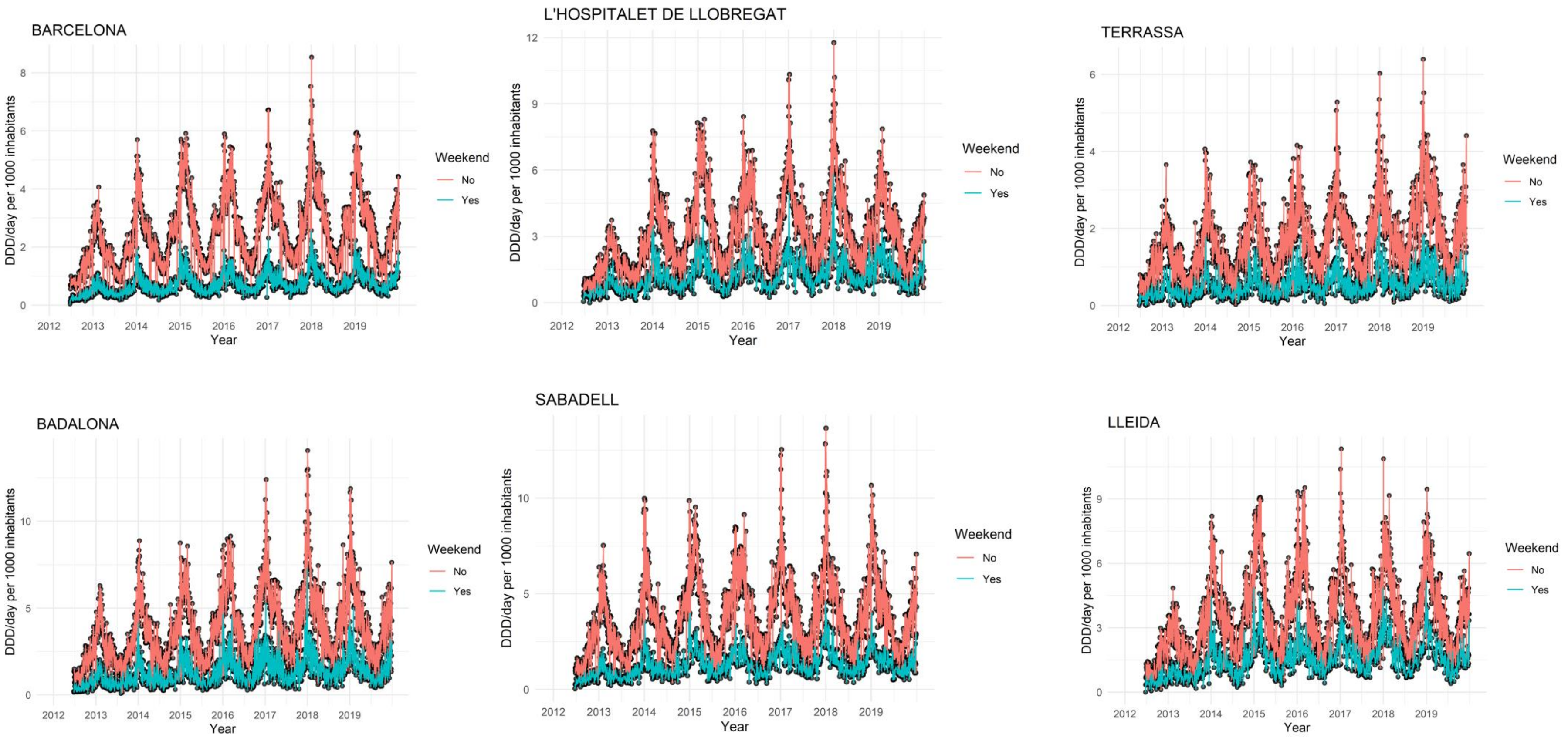

© 2024 Abelenda-Alonso G et al. *JAMA Network Open.*

eFigure 2 (continued). Temporal Series of DDD per 1,000 Inhabitants-Day for the 11 Cities Studied

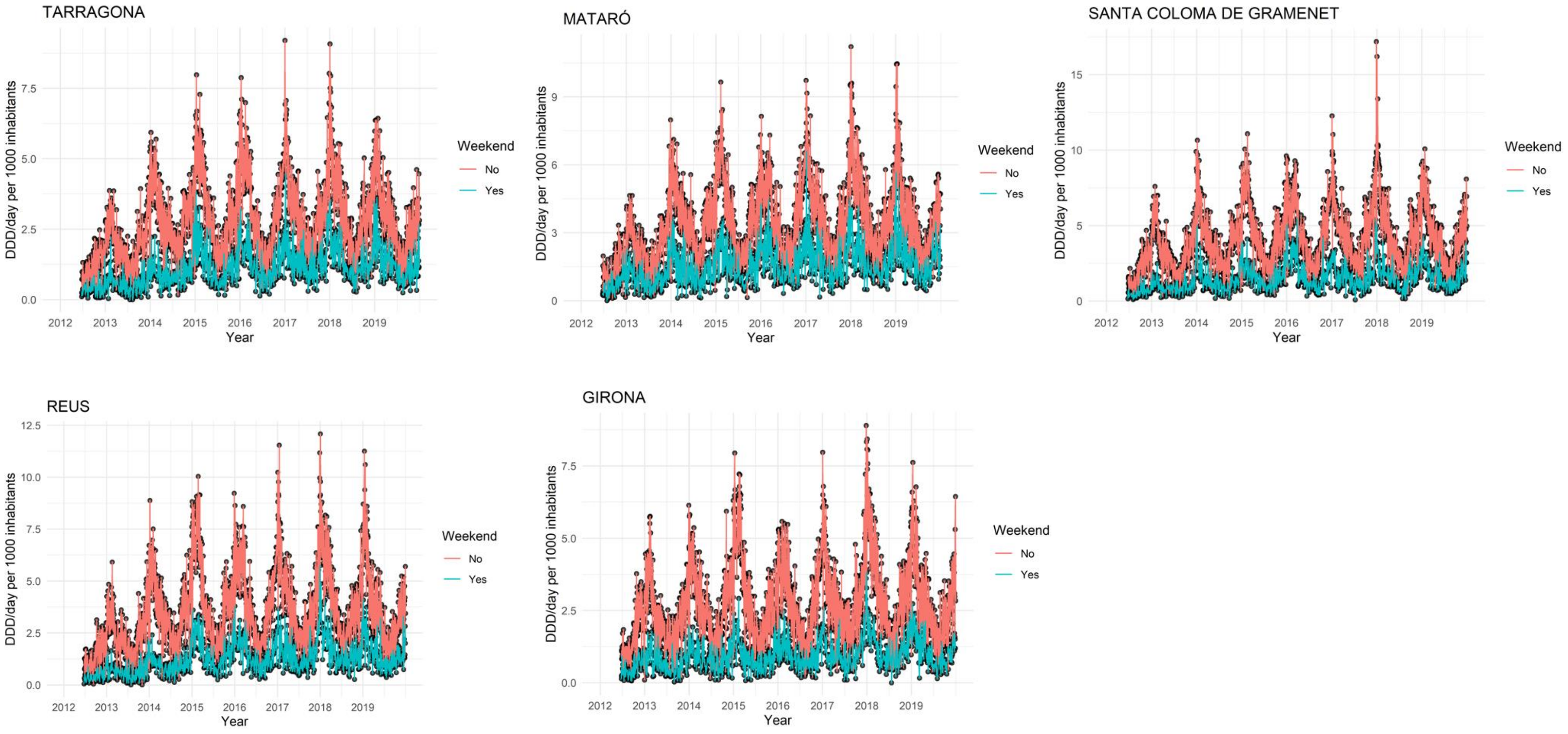

© 2024 Abelenda-Alonso G et al. *JAMA Network Open.*

eTable 5. Historical Series of Potential Nonlinear Weather-Related Confounding Factors

	Mean temperature (ºC)		Relative humidity (%)		Rainfall (mm)	
**City**	**Median [Q1;Q3]**	**n (%)**	**Median [Q1;Q3]**	**n (%)**	**Median [Q1;Q3]**	**n no-zeros (%)**
BARCELONA	17.7 [13.4; 23.29]	2,748 (100%)	64.01 [56.27; 71.03]	2,748 (100%)	0.06 [0.01; 0.20]	582 (21.18%)
L'HOSPITALET DE LLOBREGAT	16.44 [12.02; 22.26]	2,748 (100%)	64.58 [57.08; 72.51]	2,748 (100%)	0.04 [0.01; 0.15]	623 (22.67%)
TERRASA	15.06 [9.84; 20.99]	2,748 (100%)	71.51 [63.58; 79.13]	2,748 (100%)	0.03 [0.00; 0.13]	716 (26.06%)
BADALONA	16.63 [12.37; 22.22]	2,746 (99.93%)	68.67 [61.11; 74.71]	2,747 (99.96%)	0.05 [0.01; 0.16]	559 (20.34%)
SABADELL	15.06 [9.84; 20.99]	2,748 (100%)	71.51 [63.58; 79.13]	2,748 (100%)	0.03 [0.00; 0.13]	716 (26.06%)
LLEIDA	14.24 [8.15; 20.55]	2,748 (100%)	69.65 [61.29; 79.33]	2,748 (100%)	0.02 [0.00; 0.09]	815 (29.66%)
TARRAGONA	17.08 [12.21; 22.76]	2,746 (99.93%)	70.83 [59.94; 77.42]	2,747 (99.96%)	0.03 [0.01; 0.14]	556 (20.23%)
MATARÓ	16.12 [11.75; 21.71]	2,748 (100%)	69.29 [61.79; 76.42]	2,748 (100%)	0.05 [0.01; 0.18]	660 (24.02%)
SANTA COLOMA DE GRAMANET	16.63 [12.37; 22.22]	2,746 (99.93%)	68.67 [61.11; 74.71]	2,747 (99.96%)	0.05 [0.01; 0.16]	559 (20.34%)
REUS	15.83 [10.85; 21.45]	2,748 (100%)	73.32 [63.24; 80.93]	2,748 (100%)	0.02 [0.00; 0.09]	761 (27.69%)
GIRONA	15 [9.15; 20.7]	2,748 (100%)	76.72 [69.16; 83.81]	2,748 (100%)	0.06 [0.01; 0.17]	717 (26.09%)

© 2024 Abelenda-Alonso G et al. *JAMA Network Open.*

eFigure 3. Map of the Studied Area Showing the Geographical Location of Each Station

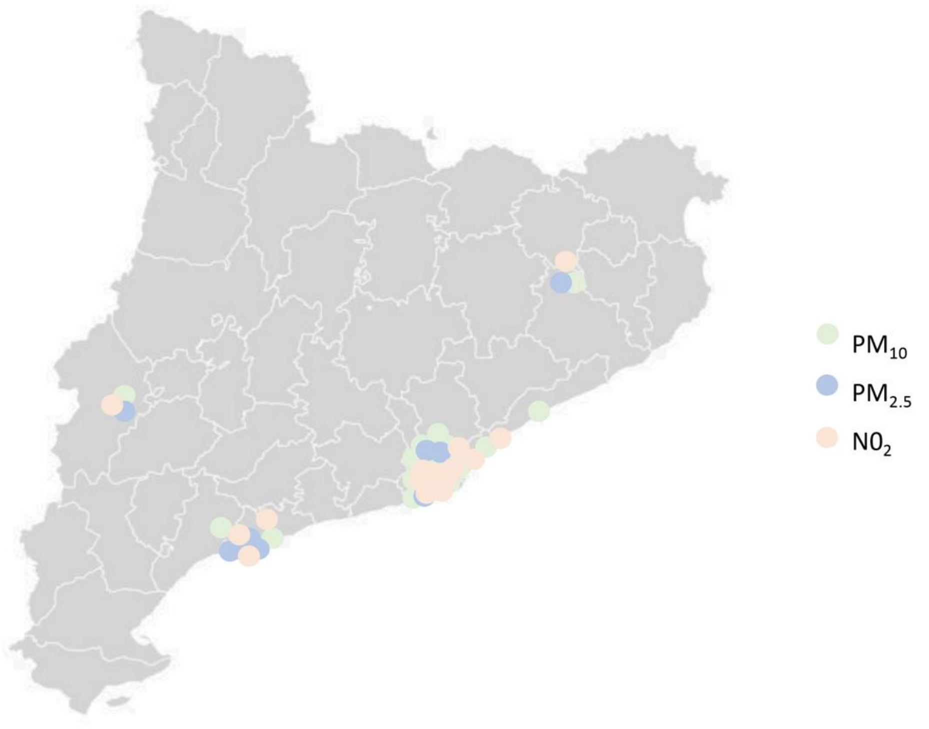

© 2024 Abelenda-Alonso G et al. *JAMA Network Open.*

eFigure 4. Study Flowchart

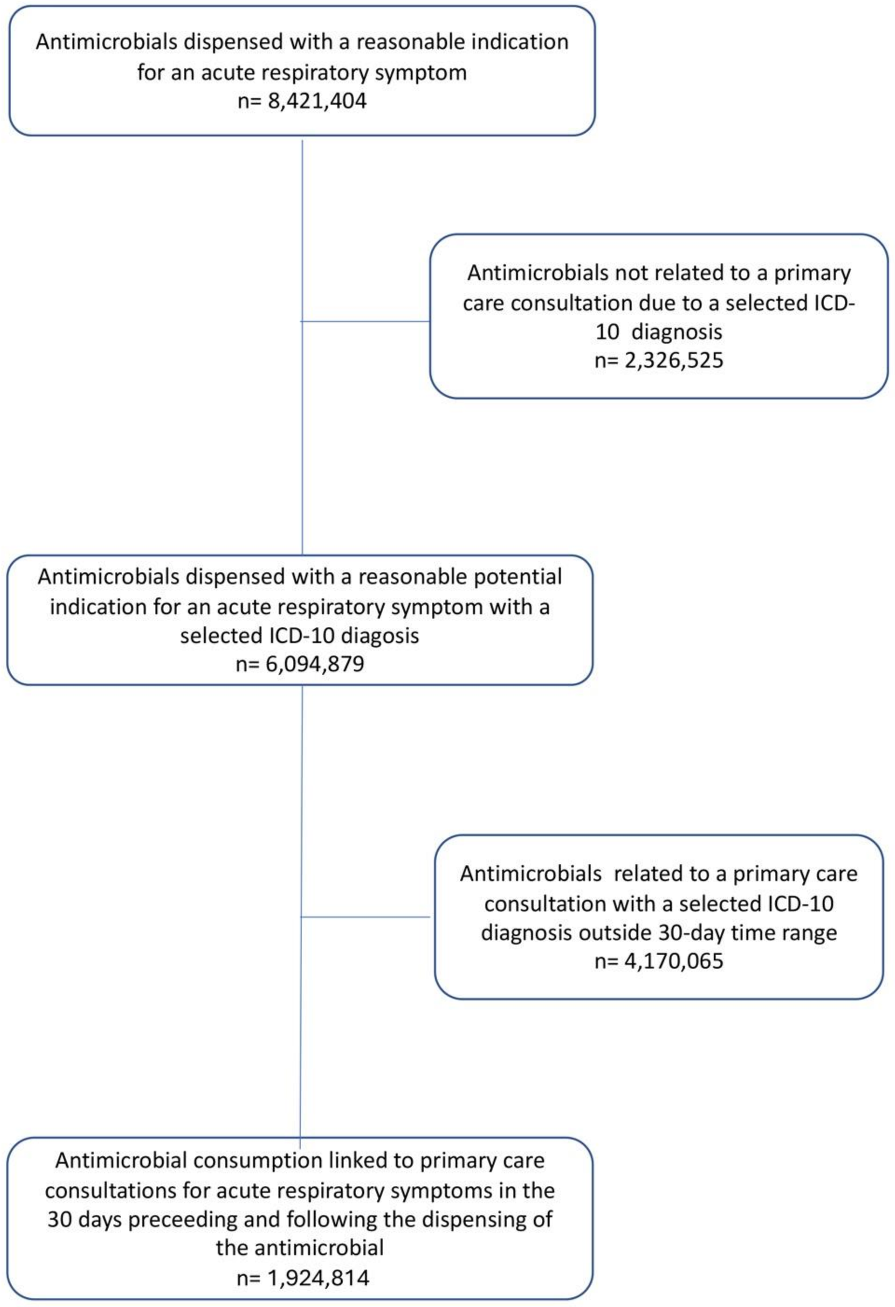

© 2024 Abelenda-Alonso G et al. *JAMA Network Open.*

eTable 6. Meta-analysis of the Estimated Relative Risks (RR [95% CI]) of an Overall Increase in Antimicrobial Consumption With a 10 μg per Cubic Meter Increase in PM_{10} for Each City Studied

City	Lag0	Lag1	Lag2	Lag3	Lag4	Lag5	Lag6	Lag7	Lag8	Lag9	Lag10	Lag11	Lag12	Lag13	Lag14
BARCELONA	1.016 [1.005;1.027]	0.995 [0.988;1.003]	0.993 [0.988;0.999]	0.997 [0.993;1]	0.999 [0.995;1.003]	1.000 [0.997;1.003]	1.000 [0.998;1.003]	1.001 [0.998;1.003]	1.001 [0.999;1.004]	1.002 [0.999;1.004]	1.002 [0.999;1.004]	1.002 [1;1.004]	1.002 [0.999;1.004]	1.002 [0.998;1.005]	1.001 [0.997;1.006]
L'HOSPITALET DE LLOBREGAT	1.009 [0.997;1.022]	0.99 [0.981;0.998]	0.993 [0.986;0.999]	0.999 [0.994;1.005]	1.002 [0.996;1.007]	1.001 [0.997;1.005]	1.000 [0.997;1.003]	0.999 [0.996;1.003]	0.999 [0.995;1.002]	0.999 [0.995;1.002]	0.999 [0.996;1.002]	1.000 [0.997;1.003]	1.001 [0.997;1.004]	1.002 [0.997;1.006]	1.003 [0.997;1.009]
TERRASSA	1.005 [0.991;1.019]	1.000 [0.989;1.01]	1.001 [0.994;1.009]	1.003 [0.996;1.009]	1.002 [0.995;1.009]	1.000 [0.995;1.005]	0.998 [0.994;1.002]	0.997 [0.993;1.002]	0.997 [0.992;1.002]	0.997 [0.993;1.002]	0.998 [0.994;1.002]	1.000 [0.996;1.003]	1.001 [0.997;1.005]	1.003 [0.998;1.009]	1.005 [0.998;1.013]
BADALONA	1.003 [0.99;1.016]	1.000 [0.991;1.009]	1.001 [0.994;1.007]	1.002 [0.996;1.007]	1.002 [0.996;1.007]	1.001 [0.997;1.006]	1.001 [0.998;1.004]	1.001 [0.998;1.005]	1.001 [0.998;1.005]	1.002 [0.998;1.005]	1.003 [0.999;1.006]	1.003 [1;1.007]	1.004 [1.001;1.008]	1.006 [1.001;1.01]	1.007 [1;1.013]
SABADELL	1.003 [0.991;1.015]	0.996 [0.988;1.005]	0.998 [0.992;1.004]	1.001 [0.995;1.006]	1.001 [0.996;1.007]	1.001 [0.996;1.005]	1.000 [0.997;1.003]	0.999 [0.996;1.002]	0.999 [0.995;1.002]	0.999 [0.995;1.002]	0.998 [0.995;1.002]	0.999 [0.996;1.001]	0.999 [0.996;1.002]	0.999 [0.995;1.003]	0.999 [0.993;1.005]
LLEIDA	1.005 [0.996;1.014]	0.994 [0.987;1.001]	0.994 [0.989;0.999]	0.997 [0.993;1.001]	0.998 [0.994;1.003]	0.999 [0.996;1.002]	0.999 [0.996;1.001]	0.999 [0.996;1.001]	0.998 [0.996;1.001]	0.998 [0.996;1.001]	0.998 [0.996;1.001]	0.998 [0.996;1]	0.998 [0.995;1]	0.998 [0.994;1.001]	0.997 [0.993;1.002]
TARRAGONA	1.011 [0.998;1.025]	1.001 [0.991;1.012]	0.996 [0.989;1.004]	0.995 [0.989;1.001]	0.996 [0.99;1.002]	0.997 [0.993;1.002]	0.999 [0.995;1.003]	1.000 [0.996;1.004]	1.000 [0.996;1.005]	1.001 [0.996;1.005]	1.000 [0.996;1.004]	1.000 [0.996;1.003]	0.999 [0.995;1.003]	0.998 [0.993;1.003]	0.997 [0.99;1.004]
MATARÓ	1.02 [1.001;1.039]	1.002 [0.988;1.016]	0.992 [0.983;1.002]	0.99 [0.982;0.997]	0.992 [0.984;1]	0.996 [0.99;1.002]	1.000 [0.995;1.005]	1.003 [0.998;1.008]	1.005 [1;1.01]	1.006 [1.001;1.011]	1.006 [1.001;1.01]	1.005 [1.001;1.01]	1.004 [0.999;1.009]	1.002 [0.996;1.009]	1.001 [0.992;1.01]
SANTA COLOMA DE GRAMENET	1.006 [0.992;1.021]	0.983 [0.971;0.995]	0.99 [0.982;0.998]	1.000 [0.993;1.007]	1.003 [0.996;1.011]	1.003 [0.997;1.008]	1.001 [0.996;1.005]	1.000 [0.995;1.005]	0.999 [0.994;1.004]	0.999 [0.994;1.004]	0.999 [0.995;1.004]	1.000 [0.996;1.004]	1.001 [0.997;1.006]	1.003 [0.997;1.009]	1.004 [0.996;1.013]
REUS	1.02 [1.005;1.034]	1.000 [0.99;1.011]	0.999 [0.992;1.006]	1.003 [0.997;1.009]	1.004 [0.998;1.01]	1.005 [1;1.009]	1.005 [1.001;1.008]	1.004 [1.001;1.008]	1.004 [1;1.008]	1.004 [1;1.008]	1.003 [0.999;1.007]	1.002 [0.999;1.006]	1.002 [0.998;1.006]	1.001 [0.996;1.007]	1.000 [0.993;1.008]
GIRONA	1.032 [1.012;1.053]	0.99 [0.975;1.005]	0.989 [0.978;1]	0.998 [0.989;1.008]	1.003 [0.993;1.012]	1.004 [0.997;1.011]	1.003 [0.998;1.009]	1.003 [0.997;1.009]	1.002 [0.996;1.009]	1.002 [0.996;1.008]	1.001 [0.996;1.007]	1.001 [0.995;1.006]	1.000 [0.994;1.006]	0.999 [0.992;1.007]	0.999 [0.988;1.009]

© 2024 Abelenda-Alonso G et al. *JAMA Network Open.*

eTable 7. Meta-analysis of the Estimated Relative Risks (RR [95% CI]) of an Overall Increase in Antimicrobial Consumption With a 10 μg per Cubic Meter Increase in $PM_{2.5}$ for Each City Studied

City	Lag0	Lag1	Lag2	Lag3	Lag4	Lag5	Lag6	Lag7	Lag8	Lag9	Lag10	Lag11	Lag12	Lag13	Lag14
BARCELONA	1.024	0.992	0.989	0.995	0.999	1.001	1.002	1.003	1.003	1.003	1.003	1.003	1.003	1.003	1.002
	[1.005;1.044]	[0.979;1.005]	[0.98;0.999]	[0.989;1.002]	[0.992;1.006]	[0.995;1.006]	[0.998;1.006]	[0.998;1.007]	[0.998;1.008]	[0.999;1.008]	[1;1.007]	[1;1.007]	[0.999;1.007]	[0.997;1.008]	[0.995;1.01]
TARRAGONA	1.031	0.994	0.992	1	1.004	1.005	1.005	1.005	1.005	1.004	1.002	1.001	1	0.998	0.996
	[1.006;1.056]	[0.977;1.011]	[0.98;1.005]	[0.991;1.009]	[0.994;1.013]	[0.998;1.013]	[1;1.011]	[0.999;1.011]	[0.998;1.011]	[0.998;1.01]	[0.997;1.008]	[0.996;1.006]	[0.994;1.006]	[0.99;1.006]	[0.986;1.007]

© 2024 Abelenda-Alonso G et al. *JAMA Network Open.*

eTable 8. Meta-analysis of the Estimated Relative Risks (RR [95% CI]) of an Overall Increase in Antimicrobial Consumption With a 10 µg per Cubic Meter Increase in NO_2 for Each City Studied

City	Lag0	Lag1	Lag2	Lag3	Lag4	Lag5	Lag6	Lag7	Lag8	Lag9	Lag10	Lag11	Lag12	Lag13	Lag14
BARCELONA	1.031 [1.023;1.038]	0.991 [0.986;0.997]	0.991 [0.987;0.994]	0.999 [0.996;1.002]	1.002 [0.999;1.005]	1.002 [0.999;1.004]	1.001 [0.999;1.003]	1.000 [0.998;1.002]	1.000 [0.998;1.002]	1.000 [0.998;1.002]	1.000 [0.999;1.002]	1.001 [0.999;1.003]	1.002 [1;1.004]	1.003 [1;1.005]	1.004 [1;1.007]
HOSPITALET DE LLOBREGAT	1.024 [1.016;1.033]	0.99 [0.984;0.995]	0.99 [0.986;0.994]	0.999 [0.995;1.002]	1.002 [0.999;1.006]	1.002 [1;1.005]	1.002 [1;1.004]	1.001 [0.999;1.003]	1.001 [0.998;1.003]	1.000 [0.998;1.003]	1.000 [0.998;1.002]	1.000 [0.998;1.002]	1.000 [0.998;1.003]	1.001 [0.998;1.004]	1.001 [0.997;1.005]
TERRASSA	1.037 [1.026;1.048]	0.992 [0.984;1]	0.99 [0.984;0.995]	0.997 [0.992;1.002]	1.000 [0.995;1.005]	1.001 [0.997;1.004]	1.000 [0.997;1.003]	0.999 [0.996;1.003]	0.999 [0.996;1.003]	0.999 [0.996;1.003]	1.000 [0.997;1.003]	1.000 [0.997;1.003]	1.001 [0.998;1.004]	1.001 [0.997;1.006]	1.002 [0.996;1.008]
BADALONA	1.02 [1.01;1.029]	0.993 [0.986;1]	0.989 [0.984;0.995]	0.993 [0.988;0.997]	0.995 [0.99;0.999]	0.996 [0.992;0.999]	0.996 [0.994;0.999]	0.997 [0.994;1]	0.997 [0.994;1]	0.997 [0.994;1.001]	0.998 [0.995;1]	0.998 [0.995;1]	0.98 [0.995;1.001]	0.998 [0.994;1.002]	0.998 [0.993;1.003]
SABADELL	1.048 [1.038;1.058]	0.983 [0.976;0.991]	0.982 [0.977;0.987]	0.996 [0.991;1]	1.001 [0.997;1.006]	1.001 [0.998;1.005]	1.000 [0.998;1.003]	1.000 [0.997;1.002]	0.999 [0.996;1.002]	1.000 [0.997;1.003]	1.000 [0.998;1.003]	1.001 [0.999;1.004]	1.003 [1;1.005]	1.004 [1.001;1.008]	1.006 [1.001;1.011]
LLEIDA	1.043 [1.029;1.058]	0.989 [0.979;0.999]	0.987 [0.979;0.994]	0.997 [0.992;1.003]	1.002 [0.997;1.008]	1.004 [1;1.008]	1.004 [1;1.007]	1.003 [1;1.007]	1.003 [0.999;1.007]	1.002 [0.998;1.006]	1.002 [0.998;1.005]	1.001 [0.998;1.004]	1.000 [0.996;1.004]	0.999 [0.994;1.004]	0.998 [0.992;1.005]
TARRAGONA	1.072 [1.053;1.091]	0.979 [0.966;0.991]	0.975 [0.966;0.984]	0.993 [0.986;1.001]	1.001 [0.993;1.009]	1.002 [0.996;1.008]	1.001 [0.996;1.006]	1.000 [0.995;1.005]	0.999 [0.994;1.005]	0.999 [0.994;1.004]	0.999 [0.994;1.004]	0.999 [0.994;1.003]	0.999 [0.994;1.004]	0.999 [0.993;1.006]	1.000 [0.991;1.008]
MATARÓ	1.041 [1.027;1.055]	0.985 [0.975;0.995]	0.985 [0.978;0.992]	0.997 [0.991;1.003]	1.002 [0.996;1.008]	1.002 [0.997;1.006]	1.000 [0.997;1.004]	0.999 [0.995;1.003]	0.999 [0.995;1.003]	0.999 [0.995;1.002]	0.999 [0.995;1.002]	0.999 [0.996;1.002]	1.000 [0.997;1.004]	1.001 [0.996;1.006]	1.002 [0.996;1.009]
SANTA COLOMA DE GRAMENET	1.025 [1.013;1.037]	0.987 [0.979;0.996]	0.988 [0.982;0.994]	0.997 [0.991;1.002]	1.000 [0.995;1.005]	1.000 [0.996;1.004]	0.998 [0.995;1.002]	0.998 [0.994;1.001]	0.997 [0.994;1.001]	0.997 [0.994;1.001]	0.998 [0.995;1.001]	0.999 [0.995;1.002]	0.999 [0.996;1.003]	1.000 [0.996;1.005]	1.001 [0.995;1.008]
REUS	1.038 [1.022;1.054]	0.993 [0.981;1.005]	0.99 [0.982;0.998]	0.998 [0.991;1.004]	1.002 [0.995;1.009]	1.003 [0.998;1.008]	1.003 [0.999;1.007]	1.003 [0.999;1.007]	1.003 [0.998;1.007]	1.002 [0.998;1.007]	1.002 [0.998;1.005]	1.001 [0.997;1.004]	1.000 [0.996;1.004]	0.999 [0.994;1.005]	0.998 [0.991;1.006]
GIRONA	1.091 [1.069;1.113]	0.995 [0.98;1.009]	0.981 [0.971;0.992]	0.992 [0.983;1.001]	0.999 [0.99;1.008]	1.002 [0.995;1.009]	1.004 [0.998;1.01]	1.005 [0.998;1.011]	1.005 [0.998;1.012]	1.004 [0.998;1.011]	1.003 [0.997;1.009]	1.001 [0.996;1.007]	0.999 [0.993;1.005]	0.997 [0.989;1.005]	0.995 [0.984;1.005]

© 2024 Abelenda-Alonso G et al. *JAMA Network Open.*

eTable 9. Meta-analysis of the Estimated Relative Risks (RR [95% CI]) of an Increase of a 10-µg per Cubic Meter in Studied Pollutants, in Antimicrobial Consumption for Acute Respiratory Symptoms in the 15 days Preceding and Following the Dispensing of the Antimicrobial

	PM_{10}		$PM_{2.5}$		NO_2	
Lag 0	1.011 [1.006-1.015]	<0.001	1.026 [1.01-1.043]	0.0019	1.041 [1.034-1.048]	<0.001
Lag 1	0.995 [0.992-0.998]	0.1837	0.992 [0.981-1.003]	1.2003	0.988 [0.984-0.991]	<0.001
Lag 2	0.995 [0.993-0.997]	0.0104	0.991 [0.983-0.999]	0.6633	0.986 [0.983-0.989]	<0.001
Lag 3	0.999 [0.997-1.001]	1.3124	0.998 [0.99-1.006]	1.2995	0.997 [0.995-0.999]	0.1310
Lag 4	1.000 [0.998-1.002]	0.7820	1.002 [0.994-1.010]	0.6920	1.001 [0.999-1.003]	0.3306
Lag 5	1.001 [0.999-1.002]	0.4842	1.003 [0.997-1.010]	0.3194	1.002 [1.000-1.003]	0.0457
Lag 6	1.000 [0.999-1.002]	0.5829	1.004 [0.999 -1.008]	0.1126	1.001 [1.000-1.002]	0.1572
Lag 7	1.000 [0.999-1.002]	0.7574	1.004 [1.00 -1.008]	0.0570	1.000 [0.999-1.002]	0.5261
Lag 8	1.000 [0.999-1.002]	0.8170	1.004 [1.00 -1.008]	0.0563	1.000 [0.999-1.002]	0.8566
Lag 9	1.000 [0.999-1.002]	0.7800	1.003 [1.00 -1.007]	0.0755	1.000 [0.999-1.001]	0.9908
Lag 10	1.000 [0.999-1.002]	0.6617	1.003 [0.999-1.007]	0.1328	1.000 [0.999-1.001]	0.9557
Lag 11	1.000 [0.999-1.002]	0.4908	1.002 [0.998-1.006]	0.3072	1.000 [0.999-1.001]	0.7444
Lag 12	1.001 [0.999-1.002]	0.3605	1.001 [0.996-1.006]	0.6215	1.000 [0.999-1.002]	0.4755
Lag 13	1.001 [0.999-1.003]	0.3224	1.000 [0.994-1.007]	0.9031	1.001 [0.999-1.002]	0.3377
Lag 14	1.001 [0.999-1.003]	0.3333	1.000 [0.991-1.008]	1.0731	1.001 [0.999-1.003]	0.2917

eFigure 5. Meta-analysis of the Estimated Risk of Increased Antimicrobial Consumption, With Interquartile Range (IQR) for Each Pollutant

A PM_{10}

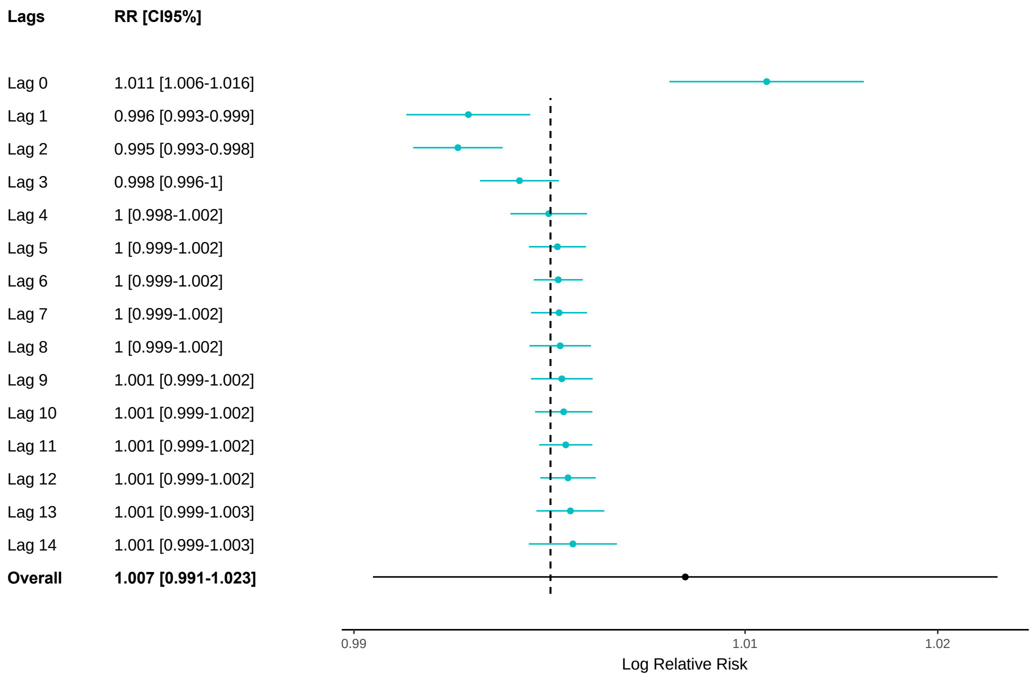

B $PM_{2.5}$

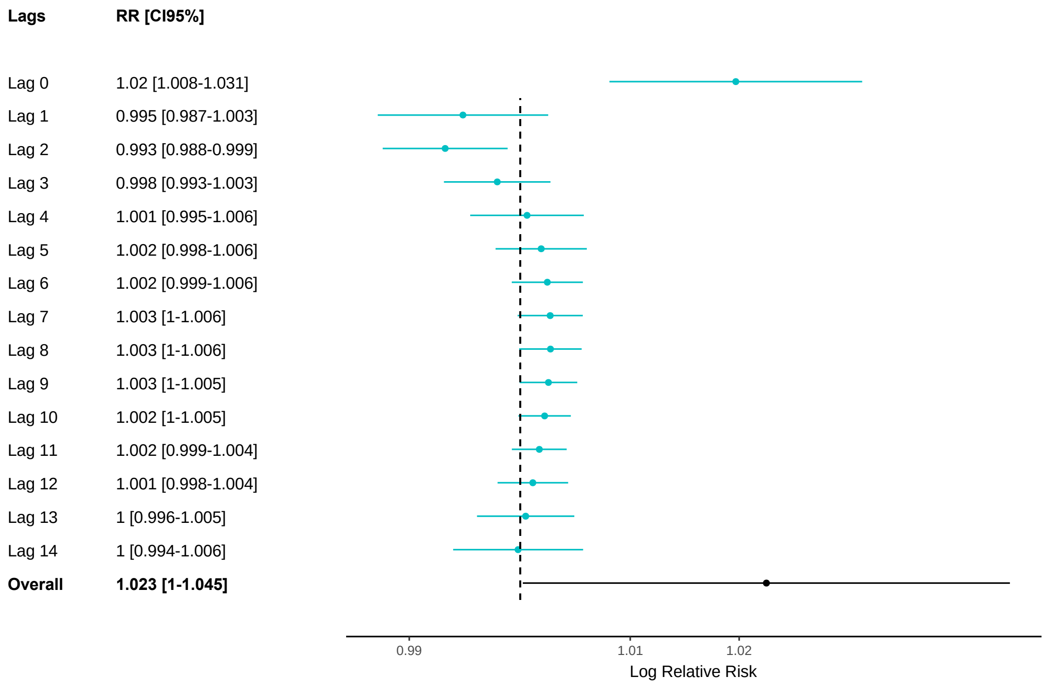

© 2024 Abelenda-Alonso G et al. *JAMA Network Open.*

C NO_2

Lags	RR [CI95%]
Lag 0	1.091 [1.074-1.108]
Lag 1	0.976 [0.969-0.982]
Lag 2	0.971 [0.965-0.977]
Lag 3	0.992 [0.988-0.996]
Lag 4	1.001 [0.997-1.006]
Lag 5	1.003 [1-1.006]
Lag 6	1.002 [0.999-1.005]
Lag 7	1.001 [0.998-1.004]
Lag 8	1 [0.998-1.003]
Lag 9	1 [0.997-1.003]
Lag 10	1 [0.998-1.003]
Lag 11	1 [0.998-1.003]
Lag 12	1.001 [0.998-1.003]
Lag 13	1.001 [0.998-1.004]
Lag 14	1.002 [0.998-1.006]
**Overall**	**1.037 [1.008-1.068]**

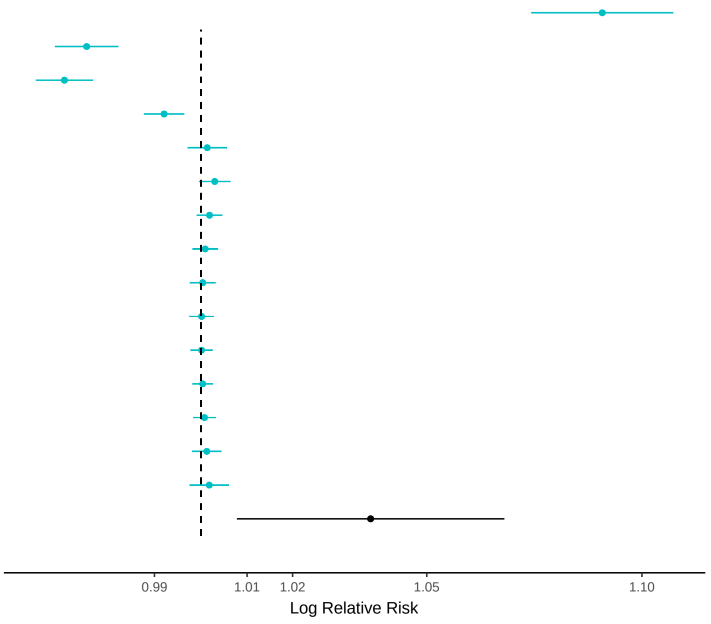

© 2024 Abelenda-Alonso G et al. *JAMA Network Open.*

eFigure 6. Heatmap for the Estimated Risk of Increased Antimicrobial Consumption, With Interquartile Range (IQR) for Each Pollutant and Each City

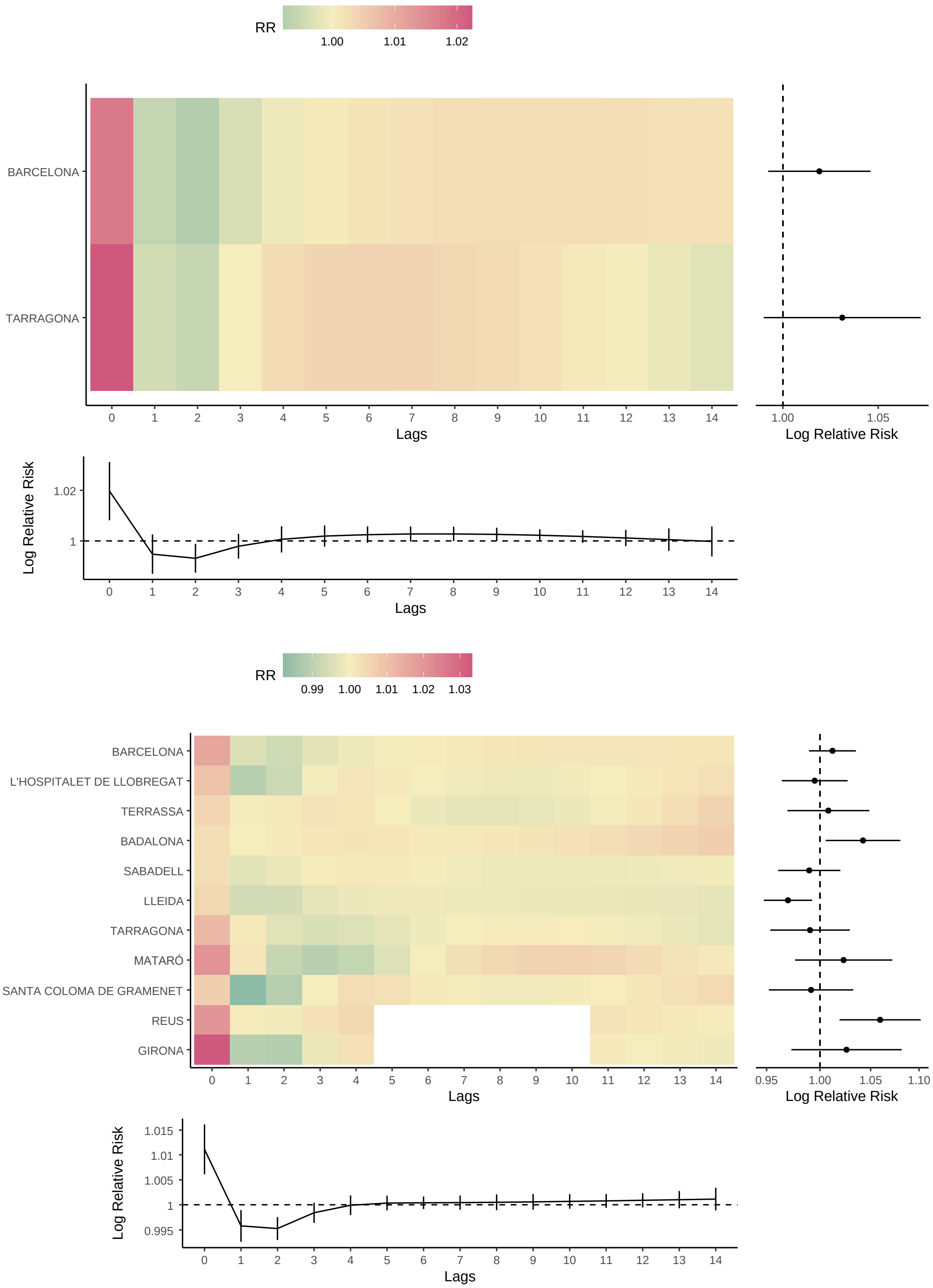

© 2024 Abelenda-Alonso G et al. *JAMA Network Open.*

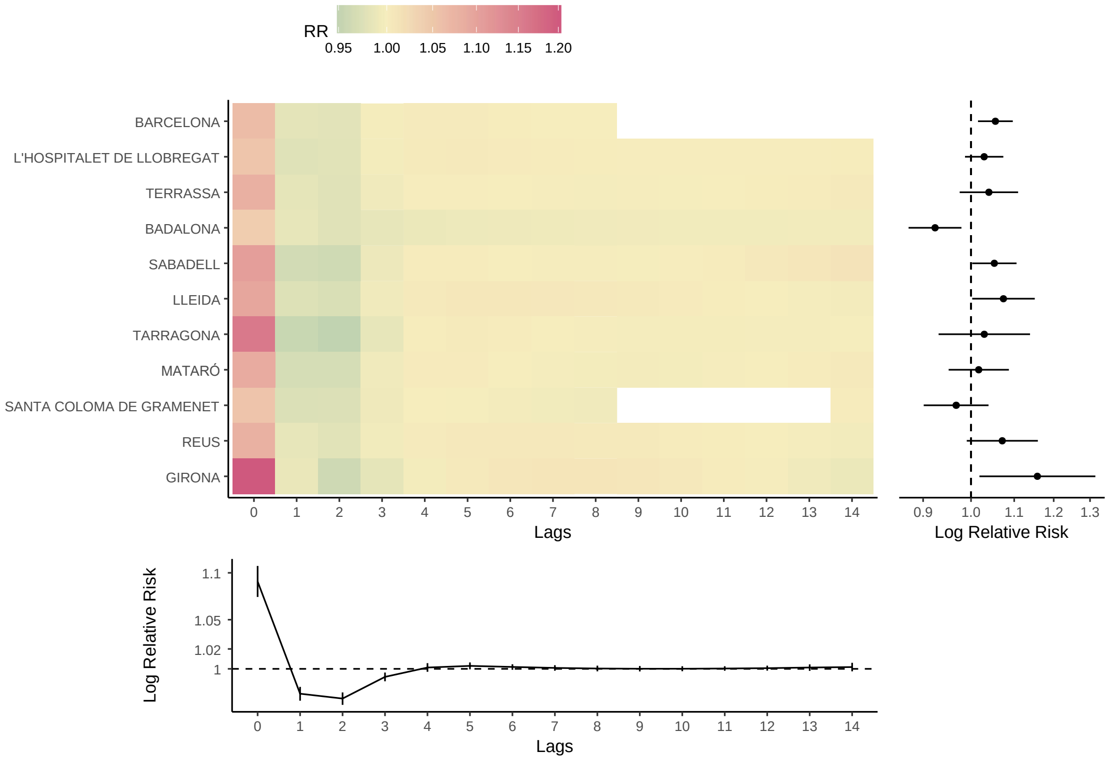

© 2024 Abelenda-Alonso G et al. *JAMA Network Open.*
