# Supplementary material for: Short-Term Exposure to Ambient Air Pollution and Antimicrobial Use for Acute Respiratory Symptoms
Source: JAMA Netw Open. 2024 Sep 6;7(9):e2432245. doi: 10.1001/jamanetworkopen.2024.32245 (PMC11380104; doi:10.1001/jamanetworkopen.2024.32245)
Supplement: Supplement 2. — Data Sharing Statement [file jamanetwopen-e2432245-s002.pdf]

Data Sharing Statement

Abelenda-Alonso. Short-Term Exposure to Ambient Air Pollution and Antimicrobial Use for Acute Respiratory Symptoms. *JAMA Netw Open*. Published September 06, 2024. doi:10.1001/jamanetworkopen.2024.32245

Data
Data available: Yes
Data types: Deidentified participant data
How to access data: Request to the corresponding author
When available: beginning date: 12-31-2025

Supporting Documents
Document types: None

Additional Information
Who can access the data: Researchers whose proposed use of the data have been approved
Types of analyses: Any reasonable research proposal
Mechanisms of data availability: With investigator support after approval (including ethics committee) of a proposal
